# Psychedelics, Sociality, and Human Evolution

**DOI:** 10.3389/fpsyg.2021.729425

**Published:** 2021-09-29

**Authors:** José Manuel Rodríguez Arce, Michael James Winkelman

**Affiliations:** ^1^Evolutionary Anthropologist, Independent Researcher, San José, Costa Rica; ^2^School of Human Evolution and Social Change, Arizona State University, Tempe, AZ, United States

**Keywords:** drug instrumentalization, evolution of religion, hominin evolution, niche-construction theory, sociality, socio-cognitive niche, psilocybin, psychedelics

## Abstract

Our hominin ancestors inevitably encountered and likely ingested psychedelic mushrooms throughout their evolutionary history. This assertion is supported by current understanding of: early hominins’ paleodiet and paleoecology; primate phylogeny of mycophagical and self-medicative behaviors; and the biogeography of psilocybin-containing fungi. These lines of evidence indicate mushrooms (including bioactive species) have been a relevant resource since the Pliocene, when hominins intensified exploitation of forest floor foods. Psilocybin and similar psychedelics that primarily target the serotonin 2A receptor subtype stimulate an active coping strategy response that may provide an enhanced capacity for adaptive changes through a flexible and associative mode of cognition. Such psychedelics also alter emotional processing, self-regulation, and social behavior, often having enduring effects on individual and group well-being and sociality. A homeostatic and drug instrumentalization perspective suggests that incidental inclusion of psychedelics in the diet of hominins, and their eventual addition to rituals and institutions of early humans could have conferred selective advantages. Hominin evolution occurred in an ever-changing, and at times quickly changing, environmental landscape and entailed advancement into a socio-cognitive niche, i.e., the development of a socially interdependent lifeway based on reasoning, cooperative communication, and social learning. In this context, psychedelics’ effects in enhancing sociality, imagination, eloquence, and suggestibility may have increased adaptability and fitness. We present interdisciplinary evidence for a model of psychedelic instrumentalization focused on four interrelated instrumentalization goals: management of psychological distress and treatment of health problems; enhanced social interaction and interpersonal relations; facilitation of collective ritual and religious activities; and enhanced group decision-making. The socio-cognitive niche was simultaneously a selection pressure and an adaptive response, and was partially constructed by hominins through their activities and their choices. Therefore, the evolutionary scenario put forward suggests that integration of psilocybin into ancient diet, communal practice, and proto-religious activity may have enhanced hominin response to the socio-cognitive niche, while also aiding in its creation. In particular, the interpersonal and prosocial effects of psilocybin may have mediated the expansion of social bonding mechanisms such as laughter, music, storytelling, and religion, imposing a systematic bias on the selective environment that favored selection for prosociality in our lineage.

## Introduction

Early hominins were omnivores that relied substantially on forest floor foods, including mushrooms (Sayers and Lovejoy, 2014). The presence of mycophagy and self-medication among both primates (Huffman, 1997; Hanson et al., 2003) and Paleolithic humans (Hardy et al., 2013; O’Regan et al., 2016) suggests hominins also incorporated fungi with bioactive properties in their diet. It is likely that psychedelic mushrooms from the genus *Psilocybe* were ingested by our ancestors since the Pliocene (beginning 5.3 million years ago [mya]), when semi-arboreal hominins intensified foraging activity on the ground (see White et al., 2009). Exposures to psychedelic fungi by australopithecines and early *Homo* during the Pleistocene (beginning 2.5 mya) are implied by their presence in African grasslands (Guzmán et al., 2014), especially growing on dung of ungulates [an important target of hominin scavenging and hunting for millions of years (Domínguez-Rodrigo and Pickering, 2003)]. Moreover, psilocybin-containing mushrooms are found on all continents (except Antarctica) and across most ecological zones (Guzmán et al., 1998; Guzmán, 2005; Froese et al., 2016), and thrive on landscapes affected by anthropic activities [e.g., woodland clearings and grazing pastures (Stamets, 1996)], indicating their widespread availability as *Homo* spread across Africa, into Eurasia, and eventually across the globe (see Antón et al., 2014)^1^.

Typical psychedelics such as psilocybin and lysergic acid diethylamide (LSD) modify fundamental brain processes that normally serve to constrain neural systems central to perception, emotion, cognition, and sense of self (Swanson, 2018). It is well established that such effects are generated primarily by the interaction of these substances with the serotonin (5-hydroxytryptamine; 5-HT) system, binding the 5-HT_2A_ receptor as partial agonists (Nichols, 2016). Psychedelic stimulation of 5-HT_2A_ receptors increases excitability of neocortical pyramidal neurons, augmenting extracellular glutamate release in the prefrontal cortex, and thereby disrupting cortical rhythmicity and large-scale brain networks (Carhart-Harris et al., 2014; Varley et al., 2020; Vollenweider and Preller, 2020). This alteration of distributed neural processes manifests as increased synaptic plasticity and entropy, as well as reduced integrity of discrete brain networks (e.g., functional disintegration of the default-mode network [DMN]) and reduced segregation between networks (e.g., increased functional connectivity between the DMN and dorsal attention network) (De Gregorio et al., 2018; Preller et al., 2019, 2020; Madsen et al., 2021). Such changes in brain activity and connectivity lead to a flexible, functionally more connected brain during the psychedelic state (Petri et al., 2014; Tagliazucchi et al., 2016; Mason et al., 2020). Further important mechanisms of action of psychedelics involve reduced thalamic filtering of interoceptive and exteroceptive information, which sustains an increased information flow to particular areas of the cortex (Vollenweider and Preller, 2020); and sensory bottom-up overflow and relaxed high-level priors (e.g., models related to self or social identity) as formulated by the relaxed beliefs under psychedelics (REBUS) model (Carhart-Harris and Friston, 2019; for further contextualization see Noorani and Alderson-Day, 2020).

The present paper suggests that these and other psychopharmacological properties of psilocybin could have had direct effects on the adaptation of early humans to their environment by enhancing their ability to live in highly social cooperative communities and participate in collaborative activities with shared goals and intentions. This human niche expanded the core of hominin sociality through collective intentionality, hyper cooperation, cultural transmission and innovation, teaching, and more recently, language (Boyd et al., 2011; Sterelny, 2012; Gamble et al., 2014; Tomasello, 2014). The emergence of these distinctively human capabilities occurs across our evolutionary history and involved a pattern of socio-cognitive niche construction predicated on a cumulative and ratcheting culture alongside substantive neurological and behavioral plasticity (Iriki and Taoka, 2012; Whiten and Erdal, 2012; Fuentes, 2015). In this context, psilocybin may have been harnessed to increase adaptability and fitness through its capacity to modulate the 5-HT_2A_ receptor mediated active coping strategy (Carhart-Harris and Nutt, 2017), which provides elevated cortical plasticity, enhanced rate of associative learning, and elevated capacity to mediate psychological transformation (Brouwer and Carhart-Harris, 2021).

Our model emphasizes effects of incidental ingestion of psilocybin-containing mushrooms as an environmental factor affecting hominin populations across millions of years of evolution. Eventually, psychedelic consumption was institutionalized in many pre-modern human societies in ritual activities focused on healing, divination (i.e., for obtaining otherwise inaccessible information), and socialization (e.g., in initiations) (Dobkin de Ríos, 1984; Furst, 1990; Schultes et al., 2001; Rätsch, 2005; Quirce et al., 2010; Leptourgos et al., 2020). In many instances, only male shamans ingested psychedelics (Harner, 1973). But in some cases they were also consumed by the general population (e.g., among the Huichol of Mexico, the híkuri cactus, *Lophophora williamsii*, is used by men, women, and children: Myerhoff, 1974). Hunters and gatherers likely learned about hallucinogenic plants as part of their detailed environmental knowledge (see e.g., Veile, 2018), and smaller scale societies placed high cultural value on the personal revelations produced (Boyer, 2019), which is attested to in the recurring mythological roles ascribed to the these mind-altering materials (Guerra-Doce, 2014, 2015).

Given the robust alterations of perception and consciousness produced by psychedelics and their medicinal and religious importance in some traditional cultures, it has been hypothesized that their ingestion influenced human evolution. McKenna (1992) proposed that psilocybin’s effects stimulating visual acuity, sexual activity, and ecstatic/visionary experiences influenced hominins’ foraging, sensitivity to community, as well as religious and spiritual concerns. He also argued the presence of psychedelics in the early human diet drove the rapid reorganization of the brain’s information-processing capacities by catalyzing the emergence of self-reflective consciousness and language. These hypotheses about human origins have received little attention and thus still need to be examined further. Moreover, they require additional development so that they can be empirically tested (e.g., using cross-cultural research methods and experimental approaches). The aim of this paper is to contribute to this task by formulating an evolutionary model of the adaptive utilization of psychedelics that properly integrates current anthropological and neuropsychopharmacological knowledge on these substances with the human evolutionary behavioral sciences.

We recognize that a simplistic version of McKenna’s account of human evolution implying that psilocybin use by itself led inevitably to the emergence of the unique cognitive, communicative, and cooperative patterns characteristic of modern human populations is most certainly false. Hominin entry into the socio-cognitive niche cannot be explained in terms of a single causal factor, a critical adaptive breakthrough (e.g., bipedality, tool-use, cooking, or even psychedelic use), but instead through positive feedback loops among various aspects of hominin life, an adaptive complex involving novel or greatly exaggerated features of our lineage (Sterelny, 2012). From this multifactorial and coevolutionary viewpoint, we propose psychedelics acted as an *enabling factor* in human adaptation and evolution. This means psychedelic use may have established positive feedback loops with core features of the evolving hominin lifeway, in part generating the coevolving dynamic that came to structure human evolution. This proposal is based on two premises:

(a) Psychedelics are serotonin analogs that preferentially activate the 5-HT_2A_ receptor subtype (Nichols, 2016), and thereby have effective medicinal applications in the treatment of stress-related conditions (Vollenweider and Preller, 2020) and significantly modulate aspects of creativity (Girn et al., 2020) and sociality (Preller and Vollenweider, 2019) that could have enhanced adaptability and fitness, especially in a knowledge-using, socially interdependent lifeway; and
(b) Psychedelic use can amplify symbolic behavior and a predisposition for collective rituals and synchronicity (e.g., by stimulating deployment of rhythmic, hermeneutical, and rhetorical activity to endure, make sense of, and communicate ecstatic and visionary experiences; Doyle, 2011) that could have transformed the social environment, and thus local selection pressures, through cultural niche construction.

**FIGURE 1:**
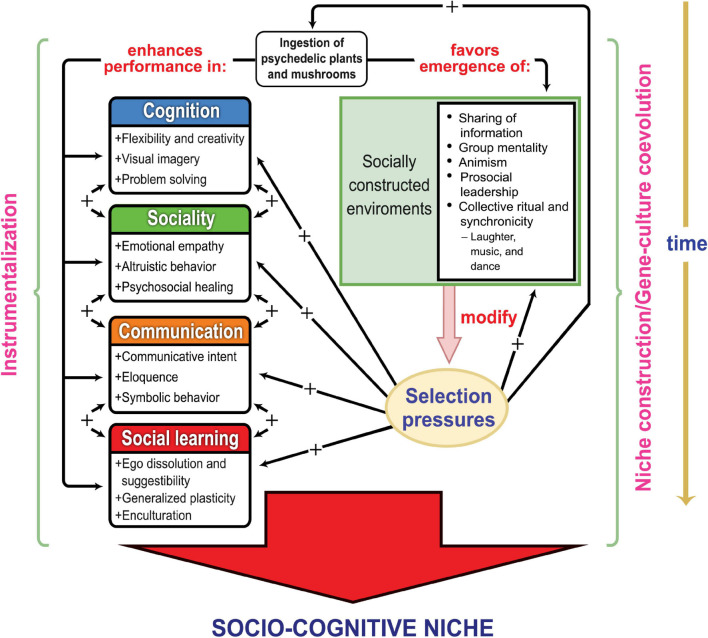
A model of psychedelics instrumentalization by early humans, and of the evolutionary consequences of its intergenerational recurrence. The left side represents the process of instrumentalization, which can occur repeatedly across the life-span of a generation of hominins. The right side represents the process of niche-construction supporting gene-culture coevolution across generations as populations construct and bequeath transformed ecological and social environments that exercise selective influences on following generations (Odling-Smee et al., 2003). The left side of the diagram portrays potential *selective advantages* conferred by psychedelic use under the socio-ecological conditions in which our ancestors evolved. The right side illustrates the process of *selective feedback* through which psychedelic instrumentalization could have enhanced the creation and evolution of the human socio-cognitive niche. The four colored boxes on the left represent the major aspects of the emerging human adaptive complex that created the socio-cognitive niche; these involve skills and processes potentially amplified by psychedelic instrumentalization, with the two-directional arrows between the boxes representing the interconnectedness of these competence realms that coevolved in creating our unique adaptation mode. The emergence and persistence of this adaptive complex across human evolution permitted the progressive construction of socially modified environments (represented by the green box at the right side of the diagram) that in turn selected for enhancements in the same underlying human propensities and capabilities (represented by arrows with a plus [+] sign) that sustained the socio-cognitive niche.

While we are definitely not proposing that psychedelics are the “missing link” in hominin evolution, we do propose that the dietary incorporation of psilocybin would have enhanced the survival and reproductive prospects of our ancestors through its incidental effects on adaptive stress-coping and enhancement of socio-cognitive dynamics. Moreover, the integration of psilocybin into ancient diet, communal practice, and proto-religious activity could have sustained feedback loops in which increases in social cognition and symbolic behavior engendered by psychedelic use selected for yet further increases in such capacities by increasing the richness and complexity of the social and semiotic environment. Psychedelics thus may have helped hominins both create and respond to a socio-cognitive niche, as hypothesized in Figure 1.

This article presents evidence for this claim. First, the main characteristics of the human socio-cognitive niche are described. Then, we examine interdisciplinary evidence supporting the hypotheses that psychedelic ingestion has deep hominin roots and that psilocybin instrumentalization conferred adaptive benefits and contributed to the human evolutionary trajectory involving advancement into a socio-cognitive niche (Barrett et al., 2007; Whiten and Erdal, 2012). The model of adaptive utilization of psychedelics presented is informed by a homeostatic perspective (Forbey et al., 2009) and the drug instrumentalization paradigm (Müller and Schumann, 2011) to explain potential selective advantages bestowed by psychedelics to hominins. The model also incorporates niche-construction (Laland et al., 2016) and gene-culture coevolutionary (Richerson et al., 2010) processes to specify how dietary and societal incorporation of psychedelics may have become evolutionarily significant by imposing a systematic bias on the selective environment that supported development of a socio-cognitive niche. The interpersonal and prosocial effects of psychedelics could have mediated expansion of social bonding mechanisms such as laughter, singing, dancing, storytelling, and religion that, in turn, accelerated the rate at which key biological components of social cognition and religiosity spread in our lineage.

## The Human Socio-Cognitive Niche

Modern humans have complex languages, sophisticated technology, intricate stores of cultural knowledge and beliefs, and an advanced theory of mind (Richerson and Christiansen, 2013; Tomasello, 2014). Early hominins may have lacked these traits (Silk, 2007), but specific selection pressures led to their acquisition in the *Homo* lineage (Schwartz and Tattersall, 2015). To explain this constellation of zoologically unusual features it has been argued that we evolved to specialize in the cognitive niche (Tooby and Devore, 1987; Cosmides and Tooby, 2001; Barrett et al., 2007; Pinker, 2010; Bertolotti and Magnani, 2017). A niche is the structural, temporal, and social context in which a species exists, defining its mode of adaptation (Fuentes, 2015). Therefore, the concept of the “cognitive niche” implies that it is mainly by thinking that humans succeed in adapting to a wider range of environments than other animals (Boyd et al., 2011). From this viewpoint, “improvisational intelligence” was selected in our lineage because the costs required to sustain it were outweighed by the benefits of the numerous solutions such intelligence could generate (Morgan, 2016).

Crucially, however, the human niche is not only about being smart: this way of life also has a cooperative core that nurtures a “deep social mind,” a way of thinking characterized by profound mental intermingling and group-mindedness (Whiten and Erdal, 2012). This novel form of socially infused thinking (Tomasello, 2014) entails unique cognitive skills and motivations for collaborating and communicating with others, such as an altruistic and egalitarian orientation and the capacity to mindread in order to enhance interpersonal coordination (Bernhard et al., 2006; Fehr et al., 2008; Heyes and Frith, 2014). Functioning in this socio-cognitive niche thus required not just intelligence and technological know-how, but more importantly the capacity for cooperation among non-kin and social learning, eventually mediated by language (Barrett et al., 2007; Whiten and Erdal, 2012). Equipped with this suit of adaptations to richly cooperative social lives (Sterelny, 2014), humans expanded across the globe, successfully adapting to a diverse range of habitats (Antón et al., 2014). We became a ‘generalist specialist,’ not only occupying and utilizing a diversity of environments, but also specializing in our adaptation to some of these environmental extremes (Roberts and Stewart, 2018).

Current understanding of the cognitive niche emphasizes that humans’ uniquely developed ability to learn from others was absolutely vital for their ecological success because it enabled the gradual accumulation of information and technologies across generations and the development of well-adapted bodies of local knowledge and complex social arrangements beyond the individual capacity to invent alone (Boyd et al., 2011; Richerson and Christiansen, 2013; Sterelny, 2014). Therefore, as has been cogently argued by Boyd et al. (2011) and Whiten and Erdal (2012), and empirically shown by Morgan (2016), the cognitive niche is eminently a social and cultural niche. We adapt not through intelligence alone but primarily through the skills, values, ideas, information, and expected modes of social interaction acquired from others in distinctively prosocial and culturally scaffolded milieus. The expansion of sociality and inter-generational cultural learning in our lineage was thus crucial for the reliable preservation of various types of expertise and the expansion of cognitive capital via cumulative cultural evolution (Sterelny, 2012, 2014).

The human socio-cognitive (or cultural) niche is simultaneously selection pressure and adaptive response (Downey and Lende, 2012). It was built and reshaped by hominins, who consequently modified the evolutionary pressures acting on them, on their descendants, and on unrelated populations sharing the same landscape (Laland and O’Brien, 2011). The various aspects of social cognition and behavior of the cognitive niche are dynamic components that established reinforcing relationships among themselves (e.g., mindreading and culture create, and in turn, are sustained through sociality), embodying an interconnected, adaptive complex that sustains our unique survival mode (Whiten and Erdal, 2012). Hence, the emergence of *Homo* was characterized by an auto-catalytic niche construction process; an iterative dynamic whereby increasing cognition, dietary quality, and cooperative behavior resulted in lowered extrinsic mortality risk and favored changes in brain size, body composition, life-history parameters, and behavioral and communicative complexity (Kaplan et al., 2000; Antón and Snodgrass, 2012; Fuentes, 2015).

The socio-cognitive niche theory invokes the undeniable practical advantages of increased cognition, sociality, communication, and social learning in order to explain the evolution of human uniqueness. Our model and the supporting evidence reviewed below suggest that the instrumentalization of psilocybin could have enhanced performance on each of these interrelated competence domains (see left side of Figure 1), potentially increasing the adaptability and fitness of our ancestors. The psychedelic instrumentalization model also proposes that psilocybin consumption had niche-constructing effects that imposed a systematic bias toward a socio-cognitive niche across the human evolutionary trajectory (see right side of Figure 1).

## Ancient Hominin Diets and the Ecology of Psilocybin-Containing Fungi

Hominin encounters with macroscopic fungi growing on the soil surface must constitute a very ancient and continual phenomenon that demanded behavioral adaptations. Fungi are widely distributed across ecozones and comprise not only valuable foods and medicines, but also highly toxic and even quickly fatal substances. Sporocarps (fungal fruitbodies) are much more abundant in the forest understory than in the middle and upper canopies where most primate species tend to live (Hanson et al., 2003). Once our hominin ancestors habitually foraged on the floors of forests and in meadows, especially in tropical areas, they recurrently encountered mushrooms. By necessity, they experimented with mycophagy and found out which species could be safely eaten as food or carefully exploited as medicine. Likewise, when psilocybin containing fungi were consumed in large enough quantities they caused dramatic alterations in perception and consciousness, drawing attention to their properties and their positive and negative effects on well-being. As a consequence, memories (and eventually cultural traditions) were formed regarding the identification of these species and the resulting effects of their ingestion. As has been hypothesized for non-human primate self-medicative behaviors (see Huffman, 1997), traditions of medicinal use of psychedelic mushrooms may have started as a result of ill, hungry hominins trying new foods during periods of extreme food scarcity, and upon recovery, associating their improved health with the new dietary item. Subsequently, local enhancement (i.e., naïve individuals having their attention drawn to species used by others) and social learning could have played a role in spreading the behavior though the group.

While incontrovertible direct evidence of psychedelic mushroom ingestion by ancient humans (e.g., dental calculus containing psilocybin mushroom tissue or spores) is lacking, there is direct evidence of the ingestion of edible mushrooms (O’Regan et al., 2016) and medicinal plants (Hardy et al., 2013) derived from analysis of dental calculus recovered from remains of humans from the Upper Paleolithic. There are 22 primate species known to eat fungi (Hanson et al., 2003), and African great apes, in particular, are known to ingest a variety of non-nutritional plants to “treat” homeostatic challenges [e.g., to aid in the control of intestinal parasites and/or provide relief from related gastrointestinal upset (Huffman, 1997)]. It thus seems highly unlikely that our hominin ancestors ignored the widespread coprophilic species of psilocybin containing mushrooms conspicuously growing on ungulates’ dung (e.g., the pantropical *Psilocybe cubensis*), especially since Plio-Pleistocene hominin activities of scavenging, hunting, and eventually domestication of bovines placed this psychedelic within the sphere of daily activities (see van Ginneken et al., 2017 for evidence and discussion regarding the similarity of migration routes of early bovines and early hominins and its implications for understanding our ancestors’ pan-African dispersal). As will be shown below, the likelihood of intentional and repeated use of psilocybin is supported by its low toxicity and by its close resemblance to the neurotransmitter serotonin, which opened up the possibility for its exploitation as a “treatment” for a significant homeostatic challenge recurrent in a socio-cognitive niche – serotonin depletion.

## Traditional Psilocybin Mushroom Ingestion and the Antiquity of Ritual Psychedelic Use

Frost (2017) reviews reports from Mesoamerican transegalitarian agricultural societies during the early contact period with the Spanish, illustrating a range of uses for psilocybin mushrooms that involve healing, spiritual, ritual, social, festive, and divinatory practices, some still reported in the 20th century (e.g., Estrada, 1989) among the Mazatecs of Oaxaca in southern Mexico. The Nahua, for instance, used these fungi in the rituals performed by Mexica (Aztec) clergy, and on a local, personal level through the assistance of medicinal/divinatory aid of *ticitls* (shamanistic healers). In the context of the official religious system, psychedelic mushroom consumption characterized notions of hospitality and ostentatiousness amongst the Mexica elite, and involved intricate ritual performances that included call and response, chanting, and dancing, as described in Hernando de Alvarado Tezozómoc’s *Crónica Mexicana* (written circa 1598) (Frost, 2017). Modern-day Mazatecs employ psilocybin mushrooms mainly to find lost items, discover hidden truths, or diagnose an ailment in the context of nocturnal rituals in which it is common for both healer and client/patient to consume the mushrooms (Estrada, 1989). This ceremony involves whistling, humming, chanting, singing, percussive artistry, ventriloquism, and dancing. To our knowledge, there are no documented foraging societies that use psilocybin fungi. This may be due, in part, to the fact that mushrooms grow mainly during the rainy season, a particularly difficult time for ethnographers to accompany the already challenging nomadic lifestyle of foragers. Possible evidence of the use of psilocybin-containing mushrooms among early Neolithic farming and herding communities may be present in rock carvings and paintings [e.g., in Africa (Samorini, 2020) and Spain (Akers et al., 2011), also see Winkelman (2019a) for review].

The utilization of psilocybin mushrooms or other psychedelic plants is documented in the ethnographic and historical literature from all cultural regions of the globe except the Insular Pacific (see Table 1 for examples). It is likely that psychedelics have been used ritually for millennia, and that this behavior has deep hominin roots. Evidence regarding the evolution of human hepatic enzymes suggests significant selection pressures were exerted on hominin populations by frequently encountered environmental chemicals, including fungal and vegetal secondary metabolites that act as stimulants, narcotics, and hallucinogens (Sullivan and Hagen, 2002; Sullivan et al., 2008). Drug consumption is not an evolutionary novelty; rather, ancient and recent exposures resulted in evolved countermeasures to tolerate them to some degree and safely metabolize them. Evidence of humans’ relationships with psychedelics during more recent times (the Holocene) is found in the archeological and paleoethnobotanical record (Guerra-Doce, 2015; Fitzpatrick, 2018; Miller et al., 2019; Samorini, 2019; Robinson et al., 2020). While the presence of psychoactive plant remains in archeological contexts does not establish their use as drugs, it is highly probable in many instances given known ethnographic analogies, artifactual associations, and iconographic interpretations (Guerra-Doce, 2014; Winkelman, 2019a; Domnauer, 2020).

**TABLE 1:** Selected societies from all over the world that employ psychedelics acting on the serotonergic system.

Region	Subregion	Culture	Species employed	Common name	Main psychoactive principles	References
Africa	West Africa	Fang	*Tabernanthe iboga*	eboka	ibogaine, ibogamine	Rätsch (2005)
Africa	Eastern Africa	Maasai	*Acacia nilotica*	olkiloriti	dimethyltryptamine (DMT), tetrahydroharman	Sobiecki (2002)
Middle East	Middle East	Iran	*Peganum harmala*	haoma	harmine, harman	Flattery and Schwartz (1989)
Asia	East Asia	Chinese	*Gymnopilus junonius*	xiàojùn	psilocybin, psilocin	Zhang and Greatrex (1987)
Europe	Southeastern Europe	Greeks	*Claviceps* spp.	kykeon	ergometrine, ergotamine	Samorini (2019)
North America	Arctic and Subarctic	Ojibwa	*Lophophora williamsii*	peyote	mescaline, pellotine	Barnouw (1950)
Middle America and the Caribbean	Central Mexico	Mazatec	*Psilocybe* spp.	ndi xi tjo	psilocybin, psilocin	Estrada (1989)
South America	Amazon and Orinoco	Tukano	*Banisteriopsis caapi*+ *Diplopterys cabrerana*	yagé	harmine, harmaline + DMT	Jackson (1983)
South America	Southern South America	Mataco	*Anadenanthera colubrina* var. *cebil*	cebil	DMT, 5-MeO-DMT	Dijour (1933)

## Homeostasis of Ingestive Behaviors and the Drug Instrumentalization Paradigm

Natural landscapes are a diverse combination of plant species that are literally nutrition centers and pharmacies with a wide range of primary (nutrient) and secondary (pharmaceutical) compounds vital to the health of plants and herbivores (Villalba and Provenza, 2007). Animals generally avoid secondary metabolites (which typically have negative physiological and behavioral consequences following their ingestion) while selecting nutrient-rich foods. However, a homeostatic perspective suggests that dietary selection is not guided simply by avoidance of plant secondary metabolites, but, in some cases, by their selection to ameliorate other challenges (Forbey et al., 2009). From this view, food selection is a quest for substances (whether nutrients or drugs) that provide homeostatic utility for the organism (Villalba and Provenza, 2007). Hence, potentially toxic secondary metabolites in fungi and plants might be actively selected by animals to achieve homeostasis. Evidence shows animals exploit the biological activity of secondary metabolites to mitigate the costs of infection by parasites, enhance reproduction, moderate thermoregulation, avoid predation, and increase alertness (Rodríguez and Wrangham, 1993; Huffman, 1997; Forbey et al., 2009).

Similarly, drug instrumentalization theory proposes that non-addictive drug use can be explained in functional terms as a purposeful adaptive process. It proposes humans and many animal species seek and consume psychoactive substances because the subsequent effects on mental states can be utilized to improve performance of goal directed behaviors (Müller and Schumann, 2011; Müller, 2020). From this viewpoint, repeated, non-addictive drug use should be modeled as a two-step process: (1) the seeking and consumption of a psychoactive drug in order to change the present mental state into a previously learned mental state, which then allows for (2) better performance of other, previously established behaviors and enhanced goal achievement (Müller and Schumann, 2011). Some instrumentalization goals proposed by the researchers include: improved social interaction; improved cognitive performance and counteracting fatigue; facilitated recovery and coping with psychological stress; and facilitation of spiritual and religious activities.

According to the homeostatic perspective, the probability of secondary metabolite exploitation is determined by the relative difference between the cost of a challenge and the toxicity of the secondary metabolite in question; the ultimate “goal” for the animal being to regulate homeostasis, achieving a balance between minimizing the cost of a challenge and minimizing toxicity (Forbey et al., 2009). We review next substantial evidence that psilocybin possesses very low toxicity and generates very few and unimportant negative side effects. This quality, in combination with the relatively high costs of the challenge it could potentially ameliorate (i.e., serotonin depletion) and the adaptive behaviors it could facilitate (discussed afterward), made psilocybin a prime candidate for instrumentalization in our lineage.

## Toxicity of Psilocybin and Associated Costs

Hagen et al. (2013) propose that plant neurotoxins currently used as drugs illustrate the necessity of their characterizations in terms of acute drug toxicity because of their fitness costs; however, the situation of psychedelics is dramatically different. Although there is a general public perception that psychedelics are dangerous, from a physiologic viewpoint they are one of the safest classes of central nervous system drugs (Nichols, 2016). Psilocybin, in particular, is exceptionally harmless. This is reflected by its high therapeutic index, 641, which is indicative of very low toxicity (Tylš et al., 2014). van Amsterdam et al.’s (2011) review of literature on psilocybin risks found that in spite of moderate acute toxicity, psilocybin has low chronic toxicity and negligible public health risk. Other public health assessments have similarly concluded that psilocybin mushrooms are the safest of all common recreational drugs (Gable, 2004; Nutt et al., 2010; Studerus et al., 2011). Moreover, psilocybin is not neurotoxic, its lethal to psychoactive dose ratio is estimated at 1000:1, it has little or no potential for creating dependence, and there is no evidence of long-term cognitive impairment (Johnson et al., 2008; Tylš et al., 2016).

Even though some adverse physical effects may occur during psychedelic action, most commonly dizziness, nausea, drowsiness, paraesthesia, blurred vision, and dilated pupils, they are relatively unimpressive even at doses yielding powerful psychological effects (Johnson et al., 2008). Higher doses are more likely to cause anxiety or fear due to feelings of ego dissolution or lack of control (Johnson and Griffiths, 2017), as well as paranoid and delusional thinking (Carhart-Harris et al., 2016b), but even exceptional overdoses don’t lead to enduring harms (Haden and Woods, 2020). Side effects such as derealization, depersonalization, long lasting unpleasant experiences (bad trips), and psychotic reactions can also occur (Strassman, 1984); however, psychological interventions are mostly sufficient and the risk of prolonged psychosis (lasting longer than 48 h) in otherwise healthy subjects after a single dose of psilocybin is rare; and in most cases, prolonged negative effects are associated with personality predispositions (Johnson et al., 2008). A large population study of 130,000 adults in the United States found no link between the use of psychedelics and suicidal behavior or mental health problems (Johansen and Krebs, 2015). Typically, when psychedelics are administered in a supportive, controlled environment (ritual or clinical setting) no severe acute or chronic adverse effects occur, and no overdose deaths have been reported after ingestion of typical doses of LSD, psilocybin, or mescaline (Nichols, 2016).

The notable potential cost of psychedelic ingestion involves the loss of cognitive structuring, opening the possibility for errors in judgment, false perceptions, distortions, and illusions that could undermine an individual’s capacity for alertness, strategic thinking, and decision-making. This specific cost (excessively “relaxed beliefs”; see Carhart-Harris and Friston, 2019), coupled with the rapid onset of mental tolerance and lack of hedonic reward (craving or withdrawal) help explain why psychedelic use is normally episodic and not compulsive, with chronic use being relatively unusual (Nichols, 2004, 2010). The ontologically shocking effects of psychedelics and their meaning-enhancing properties is likely why their use commonly occurs in engineered social contexts (e.g., in intense and immersive shared experiences consisting of multimodal performances of music, ritual, and dance: Sterelny, 2018; Winkelman, 2021c; also see St John, 2006). These factors of set (i.e., intentions, mood state, and expectations) and setting (i.e., context of ingestion, involving all sensory modes, social environment, and the set of those present) provide for protection of the psyche and integration of the experience (Dobkin de Ríos, 1984; Hartogsohn, 2016; Lifshitz et al., 2018). It seems human ancestors learned to employ psychedelics in specific contexts and in conjunction with certain “protective” behaviors that allowed them to minimize and endure negative effects (costs) and maximize and counter exploit certain qualities to maintain homeostasis and manage the challenges of group living.

## Psychedelic Self-Medication as a “Treatment” for Serotonin Depletion

From a homeostatic perspective, increased fitness can potentially result from consumption of psychoactive plants containing compounds that chemically resemble endogenous signaling molecules, especially when internal signaling functions are compromised [e.g., due to deficiencies in dietary precursors in marginal environments (Sullivan and Hagen, 2015)]. Our proposal is that the incidental ingestion of psilocybin and other psychedelic secondary metabolites that have very low toxicity and structurally resemble the neurotransmitter serotonin (5-hydroxytryptamine; 5-HT) provided a “treatment” for 5-HT depletion, a costly challenge likely recurring throughout advancement into a socio-cognitive niche (see e.g., Young and Leyton, 2002; Wood et al., 2006). Consequent to this self-medicative behavior was the development of cultural traditions of psilocybin use to ritually and symbolically exploit its salutogenic, sociality expanding, and cognitive enhancing effects (see below).

Hominin evolution occurred in settings of strong climatic and environmental variability (Potts, 2013) and involved an increasing interdependence and reliance on intelligence, cooperation, and learning from others (Sterelny, 2012). This dynamic inevitably placed a higher strain on the serotonergic system given its involvement in facilitating stress relief and mental flexibility (Carhart-Harris and Nutt, 2017; Nilsson et al., 2019) by regulating perception, cognitive function, mood, memory, and social behavior (Berger et al., 2009; Friedman, 2018; Tricklebank and Daly, 2019).

Humans cannot produce the amino acid tryptophan, precursor in the biosynthesis of 5-HT, and must obtain it through their diet (Friedman, 2018). Given early hominins’ diets low reliance on tryptophan-rich foods such as seeds, nuts, red meat, and fish (Hublin and Richards, 2009; Ungar and Sponheimer, 2011) they certainly faced deficits of this essential amino acid with the potential to perturb homeostasis through lowered levels of 5-HT. Under such circumstances, tryptamine psychedelics (e.g., DMT, psilocybin) could have provided an ideal substitute for a fundamental bioactive compound that is hard for the body to produce, effectively mimicking 5-HT’s structure and function (Nichols, 2016). Self-medication with psilocybin mushrooms would have ameliorated the costs associated with impairment of serotonergic neural signaling, involving depressed mood (Jenkins et al., 2016), increased stress vulnerability (Sachs et al., 2015), and cognitive inflexibility (Kanen et al., 2020).

5-Hydroxytryptamine moderates anxiety and stress, promotes patience and coping, and under conditions of increased environmental volatility, opens a window of plasticity for greater adaptation (Branchi, 2011; Miyazaki et al., 2012; Carhart-Harris and Nutt, 2017). Therefore, the brain displays two different serotonin-mediated responses to adversity: a default response involving a passive coping strategy (i.e., tolerating a source of stress) mediated by 5-HT_1A_ receptor signaling; and an active coping strategy that provides an enhanced capacity for change that is mediated by 5-HT_2A_ receptor signaling (Carhart-Harris and Nutt, 2017). Interestingly, serotonergic psychedelics preferentially engage the 5-HT_2A_ receptor signaling pathway, functionally modulating its activity (Nichols, 2016; Carhart-Harris and Nutt, 2017). Psilocybin thus stimulates a system that evolved to mediate rapid and deep learning when faced with environmental demands for change (Brouwer and Carhart-Harris, 2021). From the homeostatic and drug instrumentalization perspective developed here, this capacity of serotonin-mimicking psychedelics to enable a hyper-plastic state that can aid psychological transformation when (actual or perceived) environmental pressures demand it (Brouwer and Carhart-Harris, 2021) helps explain why ritualized psychedelic consumption became central to group healing, decision-making, management of ecological relations, and creation of individual and social identity in many premodern societies (Rätsch, 2005; Guerra-Doce, 2014, 2015; Kennedy, 2014).

The negative impact of 5-HT depletion on fitness likely increased as hominization ensued given an escalating dependence on cognitive skills for an intensively cooperative and collective life (Dunbar, 2014; Gamble et al., 2014). Under these conditions of demands for functioning in a socio-cognitive niche, higher-order executive tasks such as social learning, working memory, and behavioral flexibility became increasingly important; this generated an increased demand for sufficient levels of 5-HT to modulate the function of the prefrontal cortex, on which these skills critically depend (see Puig and Gulledge, 2011). Considering the costly nature of 5-HT production, its key role in hominin adaptive brain function and behavior, and the increasing selection pressures for sophisticated social cognition skills for participation in the socio-cognitive niche, it is reasonable – perhaps inevitable – that early hominins actively pursued available exogenous chemical analogs of 5-HT.

Importantly, as meat became a more pervasive item in later hominins’ diet, it is likely that tryptophan deficits were less common since it is present in high quantities in most protein-based foods (Friedman, 2018). This means that if psychedelics were initially used among hominins and archaic human species to “treat” 5-HT depletion, reliance on this self-medicative behavior may have become less important as our human ancestors’ diet progressively included seeds and nuts, as well as larger quantities of meat from large animals and fish. Thus, while psychedelics may have entered hominin evolution via their role as a “treatment” for 5-HT depletion, once full “migration” into a socio-cognitive niche was complete (which involved establishment of a foraging strategy extended to large mammalian prey) their ingestion was likely sustained because of the additional adaptive benefits their ritual and symbolic instrumentalization could confer to humans properly (see below). In other words, while tryptophan deficiency was likely no longer an issue among hunter-gatherer societies or prehistoric horticultural/agricultural societies, the premium placed on cognitive and social functions by the socio-cognitive niche meant there was still a place for counter exploiting psychedelics’ effects in our lineage.

## Psychedelic Instrumentalization in the Human Socio-Cognitive Niche

Entry into the socio-cognitive niche involved increasing cognition, sociality, communication, and social learning. Figure 1 summarizes a model of how these major aspects of the emerging human adaptive complex were potentially enhanced by incidental psychedelic ingestion and periodic psychedelic instrumentalization. The model suggests psilocybin would have amplified the requisite capacities for increasingly complex social interaction and a suite of cognitive abilities supportive of the socio-cognitive niche, including aspects of creativity, non-verbal and linguistic expression, and suggestibility (left side of Figure 1). These effects could have facilitated general problem solving, cooperative foraging, ritual healing, conventional representation and symbolization (including myth and identity formation), and enculturation practices (e.g., rites of passage). The following sections integrate current understanding of the socio-cognitive niche with recent psychedelic research (mainly controlled experimental studies in humans, both in clinical populations and healthy volunteers) to illustrate how psychedelics could have been adaptively employed by our ancestors. We focus on four interrelated psychedelic instrumentalization goals: management of psychological distress and treatment of health problems; improved social interaction and interpersonal relations; facilitation of collective ritual and religious activities; and enhanced group decision-making.

### Management of Psychological Distress and Treatment of Health Problems

Foraging is not a safe activity; it leads to significant mortality and morbidity. Even cooperative hunting, for example, is accident prone, attacks by wounded animals being paramount (Klein, 1999). Human ancestors suffered from infectious pathogens (e.g., bacteria, viruses, parasites) and social stress management imposed pressure on their time budget as group size increased (Gamble et al., 2014). We suggest under these conditions of disease and stress, psychedelic use could have improved stress management, healing, and well-being. Psychedelics can facilitate adaptive stress coping via upregulation of 5-HT_2A_ receptor functioning (Carhart-Harris and Nutt, 2017), bringing about a “pivotal mental state” characterized by an enhanced rate of associative learning and the potential for the mediation of stress through psychological or cognitive transformations (Brouwer and Carhart-Harris, 2021). These effects in enhancing active coping strategies illustrate a core aspect of psilocybin’s potential contributions to hominin adaptability and fitness.

Psychedelic abatement of psychological distress can aid in the treatment of mental illness. In contemporary clinical contexts, psychedelics have effective psychiatric applications, particularly in the treatment of stress-related disorders (dos Santos et al., 2016; Garcia-Romeu et al., 2016; Carhart-Harris and Goodwin, 2017; Johnson and Griffiths, 2017; Goldberg et al., 2020a; Luoma et al., 2020; Reiff et al., 2020; Vollenweider and Preller, 2020; Carhart-Harris et al., 2021; De Gregorio et al., 2021a; Inserra et al., 2021). Winkelman and Sessa’s (2019) edited volume contains reviews of clinical evidence showing the therapeutic effectiveness of psychedelics in the treatment of various health conditions, including anxiety, trauma, treatment-resistant depression, as well as personality, inflammatory, and autoimmune conditions (also see Szabo, 2019; Thompson and Szabo, 2020). The immersive experiences engendered by high doses of psychedelics are often attributed deep personal meaning, and a growing body of theoretical and empirical work shows they can have persisting beneficial effects on well-being and psychosocial functioning (Kuypers et al., 2016; Sweat et al., 2016; Bouso et al., 2018; Haijen et al., 2018; Carhart-Harris and Friston, 2019; Kuypers, 2019; Mason et al., 2019, 2021; Preller and Vollenweider, 2019; Barrett et al., 2020; Girn et al., 2020; Goldberg et al., 2020b; Yaden and Griffiths, 2020). There are also hints that lifetime psychedelic use is associated with markers of physical health (self-reported overall health, body mass index, and heart condition and/or cancer in the past 12 months: Simonsson et al., 2021).

Most pre-modern societies considered illness to be caused by supernatural and spiritual agents (Schultes et al., 2001; Rätsch, 2005); psychedelics can contribute to cures because they produce spiritual experiences and a sense of control over preternatural realms (Dobkin de Ríos, 1984; Furst, 1990; Winkelman, 2010). Many shamanistic healing traditions use psychedelics to facilitate an experience of contact between the ritual specialist and supernatural beings/realms, inducing visions that provide knowledge about the causes of the condition afflicting the patient and proper treatment, or allowing healers to confront and combat a disease through symbolic battles with its cause (Rivier and Lindgren, 1972; Harner, 1973; Dobkin de Ríos, 1984; Ferreira Júnior et al., 2015).

Psychedelics’ imagery-inducing (de Araujo et al., 2012), meaning-enhancing (Hartogsohn, 2018), and contextual effects (Carhart-Harris et al., 2018b) can play an important role in boosting imagination, the placebo effect, and hypnotic suggestibility, thereby favoring salutogenesis through psychoendoneuroimmunological processes (Ray, 2004). Psychosocial healing is a component of human cooperation that comprises empathy, mirroring, emotional contagion, self-regulation, and mentalizing; it also recruits symbolic processes requiring shared meanings of symbols (Kohrt et al., 2020). Thus, besides the psychedelic substance, other ritual elements (e.g., cultural expectations, mimetic enactments, verbal displays, songs, and dances) also serve an important function in enabling healing (Winkelman, 2008, 2019b, 2021a,c; Uthaug et al., 2021).

The fact that psychedelics induce an experience of well-being may have favored learning of their use by humans (Johns, 1990). Rodriguez et al. (1982) suggest hallucinogenic plants were initially used for the treatment of diseases due to their antiparasitic properties. As argued by Ferreira Júnior et al. (2015), the overlap between medicinal and hallucinogenic uses may indicate that the initial consumption of a plant for medicinal purposes lead to the discovery of its use as a hallucinogen. This is consistent with our view that psychedelics were initially ingested because they could provide homeostatic utility.

### Improved Social Interaction and Interpersonal Relations

Humans, like all primates, are intensely social. The human niche encompasses face-to-face interactions within social groups, interactions among social groups, and complex social dynamics at both group and larger community levels (Fuentes, 2015). Human ancestors faced the adaptive problem of maintaining the cohesion of large social groups in the face of the centrifugal forces created by the stresses of group living. Primates solve this problem by developing intense forms of commitment to each other through close physical proximity and the use of touch (e.g., licking and other social grooming behaviors [Dunbar, 2010]). Hominins evolved larger group sizes (100–200 individuals) by developing indirect ways (i.e., without physical contact) of triggering endorphin activation that produces community bonding (Dunbar, 2014; Gamble et al., 2014). In sequential order, these extended grooming behaviors involved laughter (a form of chorusing), singing (without words), dancing, storytelling, and more recently, religion – activities that stimulate the endogenous opioid mechanisms that enhance the sense of being bonded with others involved (Pearce et al., 2015; Tarr et al., 2015; Dunbar et al., 2016; Charles et al., 2020). The enhanced emotional ties provided human groups with a higher degree of cohesiveness and stability through time, enhancing various forms of cooperation.

We propose that in this context, psychedelics’ effects were harnessed to modulate the strength and quality of social bonds. Ingestion of psilocybin induces euphoria, involuntary grinning, uncontrollable laughter, giddiness, playfulness, and exuberance (Preller and Vollenweider, 2016); it also enhances engagement with music (Kaelen et al., 2018) and eloquence (Doyle, 2011). This means psilocybin ingestion would have amplified sociality long before the emergence of religious rituals. Once archaic humans developed religious and spiritual concerns (see following section on *Facilitation of collective ritual and religious activities*), psilocybin would have become even more useful given its intrinsic ability to produce mystical-type experiences involving the dissolution of self-boundaries and a sense of unity with others (Griffiths et al., 2006, 2011). Therefore, psychedelic use increased participation in the emerging niche in which sociality enhancing experiences such as playing and laughing, singing and dancing, fantasizing and telling stories, and participating in religious rituals became commonplace activities. Collective use of psychedelics may have thus enriched social life and bolstered hermeneutical and rhetorical activity, enhancing management of group tension (through emotional catharsis) and strengthening social bonds (by triggering the endorphin system), ultimately facilitating complex sociality and communication in the ever-larger human groups.

Recent studies show that psychedelics can modify a range of social behaviors and cognitive processes, having pro-social effects (Table 2; also see Preller and Vollenweider (2019) for a recent review of experimental and controlled studies in humans). Psilocybin has been shown to modulate different objective measures of social cognition; most importantly, it increases empathy for positive emotions (Pokorny et al., 2017) and reduces recognition and processing of negative emotional faces (Schmidt et al., 2013), which facilitates social approach behaviors and thus social interaction (Preller and Vollenweider, 2019). Psilocybin also increases altruistic behavior: employing the Ultimatum Game, Gabay et al. (2018) found that it reduced costly punishment by increasing the participants’ concern for the outcome of interacting partners. Furthermore, psilocybin shifts emotional biases away from negative toward positive stimuli (Kraehenmann et al., 2016), and a single high-dose experience can engender measurable and long-lasting changes in socially oriented aspects of personality, such as increases in the dimensions of Openness and Extraversion (MacLean et al., 2011; Bouso et al., 2018; Erritzoe et al., 2018).

**TABLE 2:** Evidence for enhanced social and interpersonal capacities during and after psychedelic exposure.

Potentially adaptive effect	Study	Summary of results	Subject population	Total number of subjects
Enhanced social approach behaviors and social interaction	Kometer et al. (2012); Dolder et al. (2016); Pokorny et al. (2017)	↑ positive mood states, ↓ recognition of negative facial expression, and ↑ behavior toward positive relative to negative cues LSD ↑ happiness, trust, closeness to others, emotional empathy, and sociality ↑explicit and implicit emotional empathy	Healthy subjects Healthy subjects Healthy subjects	17 (6 females) 40 (20 females) 33 (15 females)
Enhanced social connectedness	Watts et al. (2017)	↓ disconnection (from self, others, and the outside world), ↓ avoidance of difficult emotions and memories, and ↑ acceptance	Patients with treatment-resistant depression	20 (6 females)
Enhanced emotional self-control and tolerance	Barrett et al. (2020)	↓ negative mood, ↑ positive mood, and ↓ amygdala response to negative affective stimuli; reflecting ↑ top-down control of emotionally conflicting information	Healthy subjects	12 (7 females)
Enhanced prosocial attitudes/behaviors and healthy psychological functioning	Griffiths et al. (2018)	Positive changes on longitudinal measures of interpersonal closeness, gratitude, life meaning/purpose, forgiveness, and altruistic behavior	Healthy subjects	75 (45 females)
Positive personality changes	MacLean et al. (2011); Erritzoe et al. (2018); Netzband et al. (2020)	↑ Openness (sustained 1 year after the session) ↓ Neuroticism and ↑ Extraversion (sustained at 3-month follow-up) Ayahuasca ↑ agreeableness and ↓ neuroticism (sustained in a 6-month follow up)	Healthy subjects Patients with treatment-resistant depression Patients with depression, anxiety, or post-traumatic stress disorder	52 (30 females) 20 (6 females) 24 (9 females)

*In all studies, psilocybin was the substance administered, unless otherwise stated.*

*↑, increased; ↓, decreased.*

The unusually high level of intragroup tolerance and cooperative communication of modern humans is explained by selection for prosociality (or against aggression), a process that has been described as self-domestication (Benítez-Burraco et al., 2020). Selection likely modulated tolerance with increased brain 5-HT levels (Hare, 2017; Raghanti et al., 2018), which is consistent with the scenario developed here in which serotonergic psychedelics provided homeostatic utility by substituting for 5-HT under circumstances in which endogenous biosynthesis and thus signaling functions were compromised. Changes in social cognition also relied on decreases in emotional reactivity supported by shifts in the hormonal and subcortical profiles (e.g., amygdala reactivity) linked to temperament, which then allowed cognitive skills to be expressed in new social situations (e.g., in teaching contexts) (Hare and Tomasello, 2005; Hare, 2017). It is thus noteworthy that a single dose of psilocybin decreases amygdala reactivity to negative stimuli and increases positive mood state (Kraehenmann et al., 2016; also see Rocha et al., 2019). Such shifts in affect and the neural correlates of affective processing can endure for several weeks beyond acute drug effects (Barrett et al., 2020). This suggests that psychedelics increased social tolerance and cohesion by inducing socially desirable mood changes from reduced neural responses to negative stimuli (Kometer et al., 2012; Mueller et al., 2017; Barrett et al., 2020; Vollenweider and Preller, 2020).

Recent work with rodents has unveiled a mechanism of action potentially underlying the prosocial effects of psychedelics. De Gregorio et al. (2021b) have demonstrated that repeated administration of low doses of LSD in mice enhances social interaction by potentiating 5-HT_2A_ and AMPA receptor neurotransmission in the medial prefrontal cortex via an increasing phosphorylation of the mTORC1 (a protein involved in the modulation of social behavior). Moreover, psilocybin has been found to increase striatal dopamine concentrations in humans, a mechanism partly underlying euphoria and depersonalization phenomena (Vollenweider et al., 1999). Striatal dopamine promotes social living and increases reward emanating from social interactions (Rilling and Sanfey, 2011). The human striatum exhibits a unique neurochemical profile involving high dopamine levels, consistent with humans’ distinctive ultrasociality (Raghanti et al., 2018). This suggests psilocybin instrumentalization could have favored a dopamine-dominated striatum personality style, which is associated with enhanced sensitivity to social cues that promote social conformity, empathy, and altruism (see Raghanti et al., 2018).

### Facilitation of Collective Ritual and Religious Activities

Rituals are socially stipulated, conventional behaviors that are critical for group social interaction; they also drive cultural transmission within and between generations (Legare and Nielsen, 2020). Rituals are very diverse and complex, but often involve synchronic movement, causally opaque action, and both euphoric and dysphoric arousal (Whitehouse and Lanman, 2014; Boyer and Liénard, 2020). Ritualized behaviors in the animal kingdom have the basic function of enhancing coordination and cooperation (Winkelman, 2009, 2019b,c). In humans, they also have other social, psychological, and instrumental functions involving, for instance, signaling commitment to others, binding group members together, and reducing individual and collective anxiety (Boyer and Liénard, 2020; Legare and Nielsen, 2020; Nielsen et al., 2020). In traditional cultures, rituals often have goals related to survival and reproductive success such as curing an illness, harming a rival, or ensuring success in hunting (Rossano, 2020). They are particularly crucial during times of transition, risk, and uncertainty in the human lifespan (Legare and Nielsen, 2020).

Religion comprises symbolically and emotionally laden beliefs and practices (e.g., rituals) regarding superhuman powers, and the institutions that maintain and transmit such beliefs and practices (Bulbulia et al., 2013). A wealth of ethnographic and experimental evidence suggests that religions forge solidarity and cooperation through various mechanisms and at different levels of social complexity (Katz, 1984; Boehm, 1993; Winkelman, 2013b, 2021b; Norenzayan et al., 2016; Skoggard et al., 2020). Early religious forms developed in the context of intense and immersive experiences of music, ritual, and dance (Dunbar, 2017, 2020), likely in combination with psychedelics (Sterelny, 2018; Winkelman, 2019b,c, 2021a,c) and other mind-altering techniques (Rossano, 2007, 2009). Dunbar (2017, 2020) suggests these shamanic type religions based on trance-dancing evolved sometime between the appearance of archaic humans (i.e., Heidelbergensians) around 500,000 years ago and the appearance of anatomically modern humans (*Homo sapiens*) around 200,000 years ago as one of a series of behaviors that humans developed to enhance social bonding by triggering the endorphin system.

Foragers mainly utilize psychedelics in shamanic rituals (Harner, 1973; Dobkin de Ríos, 1984; Winkelman, 2010, 2013a) indicating a key aspect of psychedelic instrumentalization was incorporation into prosocial contexts involving synchronic activities (e.g., ritual, drumming, dancing, and singing) that were the precursors to shamanism (Winkelman, 2011a,b, 2019b, 2021c). The prosocial and interpersonal effects of psychedelics (see Table 2) likely supported the “collective effervescence” (*sensu* Durkheim, 1995) and the sense of “communitas” (*sensu* Turner, 1969) during our ancestors’ rituals, religious ceremonies, and secular celebrations, thus aiding activities that allowed individuals to reaffirm their common identity and their connectedness within the social order. Recent work by Kettner et al. (2021) lends support to this hypothesis, showing that communitas – an intense sense of togetherness and shared humanity – mediates enduring increases in psychological well-being and social connectedness following psychedelic use in ceremonial retreats.

Since pre-modern societies typically conceptualized psychedelics as entheogens [i.e., as gateways to a spiritual or religious experience and/or communication with the spirit worlds (Winkelman and Hoffman, 2015)] that provide sacred knowledge and power, they have to be understood as a source of inspiration of primordial magico-religious impulses. Importantly, controlled studies show that psychedelics reliably produce mystical-type experiences involving self-loss and a sense of awe and connectedness (Griffiths et al., 2006, 2011, 2018), as well as a range of anomalous experiences (e.g., synaesthesia, out-of-body and near-death experiences, entity encounters; see Luke, 2020; also Strassman, 2001; Winkelman, 2018) that are commonly interpreted as spiritual interactions in pre-modern cultures. Psilocybin occasioned mystical experiences produce enduring beneficial changes, such as trait-level increases in prosocial attitudes and behaviors (Griffiths et al., 2018) and in the personality domain of Openness (MacLean et al., 2011). Moreover, both psychedelic mystical experiences and entity encounters have profound and sometimes lasting effects on beliefs and worldviews (Griffiths et al., 2011, 2019; Davis et al., 2020; Lutkajtis, 2020).

Generally, in traditional smaller scale societies psychedelics are employed in socially oriented settings including healing rituals, rites of passage, initiation into secret societies and cults, and multi-group gatherings (Dobkin de Ríos, 1984; Rätsch, 2005). In these contexts, psychedelic use is carefully programmed and orchestrated by the ritual specialists to produce experiences of a confirmatory nature (Noorani and Alderson-Day, 2020), in the sense that they reinforce a set of socially situated expectations established before entering the altered state (e.g., that a cure will be effected through shamanistic magical intervention, or that initiatic contact with the ancestors will be achieved). Cross-culturally, ritual specialists leverage collective, socially bonding mythic narratives and coordinated, mixed modality performances of entrained ritual or dance to provide structuring during ego-dissolution and to evoke culturally expected visions through expressive dimensions of ritual (Dobkin de Ríos, 1984; Winkelman, 2002, 2015, 2021c; Rodríguez and Quirce, 2012). Thus, for instance, among the Tukano from the Colombian Amazon, psychedelic yagé (*Banisteriopsis caapi* + *Diplopterys cabrerana*) is administered during the Yuruparí dance rites, an ancestor ceremony for initiation of young men into adult male society (Hugh-Jones, 1979; Jackson, 1983). This intense, self-defining experience involves dancing that is interspersed with periods during which tobacco, coca, manioc beer, and yagé are consumed and myths are chanted in unison. During this ritual, young boys are supposed to enter into controlled and voluntary contact with the beginning and source of life, the (other-)world of myth, in order to gain visionary knowledge by assuming the identity of the *He* People or first ancestors (Reichel-Dolmatoff, 1971; Hugh-Jones, 1979).

Traditional enculturation rituals such as the one just described involve a “socialization of hallucinations” that involves the education of attention, the categorization of perceptions, and the shaping of emotions and expectations (Dupuis, 2021). Crucially, as argued by Dupuis (2021: 10), “Insofar as psychedelics are able to produce perceptions whose phenomenological content is strongly influenced by culture, their noetic property may enhance the significance and attribution of the reality of cultural worldviews as metaphysical, ontological, or supernatural claims… these two properties make hallucinogenic substances powerful potential vectors of cultural transmission.”

Current neuroscientific understanding of the effects of psychedelics suggests they can potentially facilitate ritual activities aimed at socialization and enculturation (such as rites of passage and initiation cults). Brain action of psychedelics involves a temporal dampening effect on activity and integrity of the default-mode network (DMN) that decreases top-down inhibition, liberating sensory and cognitive bottom-up information flow, thereby increasing the richness of subjective experience (Carhart-Harris et al., 2012, 2014; Carhart-Harris and Friston, 2019). DMN roles in self and social cognition (Spreng and Andrews-Hanna, 2015; Fingelkurts et al., 2020) are also compromised, with an inhibition or reduction of personhood and agency that leads to enhanced cognitive flexibility and emotional lability (Carhart-Harris and Nutt, 2017; Carhart-Harris and Friston, 2019). In this liminal state, suggestibility, sensitivity to context, and imagery are all heightened (Carhart-Harris et al., 2015, 2018b; Kometer and Vollenweider, 2016). Moreover, this “pivotal mental state” (Brouwer and Carhart-Harris, 2021) involves a generalized malleability, in the form of enhanced synaptogenesis and neural plasticity (Ly et al., 2018), as well as low-level learning and extinction learning (Carhart-Harris and Nutt, 2017) that can further aid self-actualization and self-editing. This destabilizing process is contained within ritual to create new meaning, mediate identity formation, and facilitate the programming of the individual into cult beliefs and cultural patterns.

### Enhanced Group Decision-Making

Hominins developed an egalitarian political system in which interdependence and the availability of lethal weapons (e.g., wooden spears and lithic points) made possible group control of leaders; consequently, group success came to depend greatly on the ability of leaders to persuade (Gintis et al., 2015). Undermining the ability of dominants to exploit others helped our ancestors replace hierarchical social dominance with a more equitable sociopolitical structure based on knowledge, conflict resolution, generosity, and status leveling mechanisms (Boehm, 1993). According to Gintis et al. (2015), the heightened social value of nonauthoritarian leadership entailed enhanced fitness for such leadership traits as the ability to form and influence coalitions and intelligence. This emerging sociopolitical system thus selected for increased cognitive and linguistic ability, which enhanced prosocial leadership skills. In this context, non-authoritarian, charismatic leaders such as shamans and other leaders with supernatural abilities flourished, specializing in palliating or preventing misfortune, providing prosocial services based on knowledge and expertise related to ritual and medicinal functions (Boyer, 2019; Garfield et al., 2020). This type of leaders played an outsized role in numerous domains beyond healing, particularly in group decision-making and problem-solving contexts (e.g., conflict and intergroup mediation, guiding hunting and group movement [Winkelman, 2010, 2013b, 2021a]).

Comparative ethnographic evidence reveals that one of the main shamanistic uses of psychedelics is for divination, i.e., for procuring otherwise unattainable information (Dobkin de Ríos, 1984; Schultes et al., 2001; Rätsch, 2005). Divination practices are required for important collective decisions in many small-scale societies (Boyer, 2020). Ingestion of a vision-inducing material is a common method to gain privileged nonempirical knowledge for decision-making (Sutton and Anderson, 2010). Psychedelic supported divination is employed for purported communication with ancestors or supernatural entities to solve diverse problems and social quandaries, to diagnose and treat illnesses, to have foreknowledge of the future, or to plan and organize subsistence-related activities (e.g., making sure that a hunting expedition will be successful) (Reichel-Dolmatoff, 1971; Dole, 1974; Myerhoff, 1974; Dobkin de Ríos, 1984; Furst, 1990; Ott, 1993). Therefore, in smaller scale societies, psychedelic use is intimately linked with strategizing and decision-making through its central role in diagnostic, forecasting, and interventionist forms of divination.

Psychedelics can afford genuine epistemic benefits even if there is no transcendent reality or all-knowing otherworldly agents (Winkelman, 2013b; Letheby, 2019). Psychedelics can offer new knowledge of old information, allowing appreciation of already known (or otherwise knowable) facts in deep, vivid, affectively and motivationally significant ways (Letheby, 2019). These knowledge-gains seem to be supported by several related psychedelic-enhanced mechanisms that include curious behavior, explorative search, structure and fact-free learning, and insight and perspective change (Carhart-Harris and Friston, 2019). Such effects were likely useful for early humans under circumstances in which immediate decisions had to be made and/or actions taken promptly despite incomplete information. Our ancestors might have been particularly drawn to the rapidly ensuing boost in cognitive flexibility, imagination, and optimism, as well as to the visual intensifications and complex imagery linked to intuitive realizations that psychedelics can facilitate (see Table 3 for a summary of behavioral and neuroimaging evidence of potentially fitness-enhancing effects of psychedelics on cognition).

**TABLE 3:** Evidence for enhanced cognitive capacities during and after psychedelic exposure.

Potentially adaptive effect	Study	Summary of results	Total number of subjects
Enriched state of consciousness	Lord et al. (2019)	Psilocybin ↑ the repertoire of brain functional network states, ↑ brain integration and neural signal complexity	15 (5 females)
Enhanced cognitive flexibility	Carhart-Harris et al. (2016a)	LSD ↑ cognitive flexibility and optimism for up to 2 weeks afterward	20 (4 females)
Heightened creativity	Family et al. (2016); Sweat et al. (2016); Mason et al. (2021)	LSD ↑ indirect semantic activation, facilitating retrieval of distant associations Naturalistic psychedelic use associated with ↑ creative problem-solving ability Acutely, psilocybin ↑ ratings of (spontaneous) creative insights and ↓ (deliberate) task-based creativity. 7 days after psilocybin, novel ideas ↑	10 (1 female) 68 (38 females) 60 (25 females)
Enhanced mental imagery	de Araujo et al. (2012)	Ayahuasca ↑ the intensity of recalled images to the same level of natural image	10 (5 females)
Enhanced ability to attribute meaning/value	Studerus et al. (2010)	Psilocybin alters the sense of meaning in percepts, e.g., ‘things around me had a new strange meaning’	327 (140 females)
Enhanced insightfulness and self-awareness	Kometer et al. (2015)	Psilocybin ↑ retrieval and reattribution of autobiographic memories	50 (22 females)

*All studies were performed with healthy volunteers.*

*↑, increased; ↓, decreased.*

Psychedelics modulate aspects of creative thought, inducing a hyper-associative, imagistic mode of thinking that operates with little logical constraints and involves making connections between relatively unrelated words and images (Girn et al., 2020). Psychedelics thus augment semantic activation (Spitzer et al., 1996; Family et al., 2016) and access to novel mental representations (Baggott, 2015), spurring unconventional associations and conceptual links that give rise to unusual thoughts. In fact, naturalistic psilocybin use has been associated with sub-acute enhancements in divergent thinking (Mason et al., 2019) and creative problem-solving ability (Sweat et al., 2016). Since reasoning about causally opaque events or outcomes – those lacking a known causal explanation – is a pervasive feature of human cognition (Legare and Nielsen, 2020), it seems fitting that certain individuals (particularly ritual leaders) in numerous cultures adopted psychedelics as instruments for inspiration and envisioning, since they provide a state of consciousness that can potentially facilitate creative generation addressing knowledge gaps.

This psychedelic-induced ‘primary process thinking’ (Kraehenmann et al., 2017) involves an increased excitability of the visual pathway (Kometer et al., 2013; Kometer and Vollenweider, 2016; Timmermann et al., 2019) and engagement of an intrinsic representational system also manifested in phantasy, daydreaming, night-time dreaming, and mystical visions (Horváth et al., 2017; Fox et al., 2018). This mode of visual mentation that likely preceded our rational, language-based consciousness supports information integration, decision making processes through presentational symbolism (involving, e.g., simulation of alternative mental scenarios), and learning (Winkelman, 2010, 2017). For millions of years, this image-based cognitive modality provided hominins with a meta-cognitive system for representation of complex relations, problem-solving, and strategic planning (Lohmar, 2010) despite it being less analytically advanced than the (logical, rule-based, and reflective) secondary process thinking (Carhart-Harris et al., 2014).

Cognitive enhancement properties of psychedelics likely derive from their modification of neural signaling, increasing system level complexity and flexibility and interconnectedness of distinct networks (Tagliazucchi et al., 2016; Lord et al., 2019). This brings about an enriched state of consciousness that spontaneously transitions between states with greater freedom and less predictably (Carhart-Harris et al., 2014; Carhart-Harris and Friston, 2019). Hence, psychedelic brain states exhibit higher signal complexity (entropy) and higher cognitive flexibility, but lower cause-effect information (Gallimore, 2015). This degradation of the brain’s ability to impose the habitual organization and categorization schemas involves a temporal disabling of the functioning of the DMN that decreases top-down inhibition and liberates bottom-up information flow to specific cortical areas, particularly via intrinsic sources such as the limbic system (Carhart-Harris and Friston, 2019; Vollenweider and Preller, 2020). According to Carhart-Harris and Friston (2019), this broadens the volume and breadth of available sensory and mnemonic content and increases the potential for ‘out of the box’ ideas, novel insights, and new perspectives. During the psychedelic state, there is also increased communication across the entire brain, which likely engages audiovisual synaesthesia and associative processing (Petri et al., 2014); and the altered integration of sensory perceptions facilitates novel experiences of self and environment, helping to reduce rigid or overly entrenched thinking patterns (De Gregorio et al., 2021a). All of this implies that psychedelic-assisted divination practices could provide access to new and unusual perspectives and innate and unconscious knowledge useful for construing judgments regarding the unknown, thereby constituting an active rhetorical coping and self-editing strategy against inevitable uncertainty.

Crucially, divination is a ritual and a tradition involving an ongoing dialog with more-than-human agents (Curry, 2010; Espírito, 2019). Cross-culturally, the belief that such supernatural agents have information people lack is widespread (Boyer, 2020). Psychedelics’ capacity to engender convincing experiences of travel to alternative worlds involving communication with autonomous entities (Winkelman, 2018; Luke, 2020) – apparently allowing a ritual specialist to channel those agents’ knowledge – likely made its ingestion a seemingly reliable procedure for obtaining inscrutable information. The robust dishabituating effects of psychedelics on behavior and their persistent rhetorical features (glossolalia and graphomania) that make discourse more attention-grabbing (Doyle, 2011) provided credible costly signals of purported direct interaction with superhuman agents, a persuasive demonstration that the diviners were not the authors of the statements they uttered. All of this reinforced “ostensive detachment,” leading people to deem such divinatory statements as less likely to be influenced by anyone’s intentions or interest, ultimately favoring efficient coordination (see Boyer, 2020).

Consequently, incipient psychedelic divination practices were mostly functional, likely taking advantage of dishabituation, creative generation, and alternative noetic and epistemic pathways in instrumental ways; at the same time, given the way they supported ostensive detachment (e.g., by reinforcing the notion that the information in question was provided by the spirits; see Winkelman, 2004), they were capable of overriding individual and collective paralysis, facilitating adaptive strategic thought and group decision making.

## Discussion

### Psychedelic Ingestion and Shamanistic Leadership in the Socio-Cognitive Niche

Shamans and other leaders with supernatural abilities that often employed psychedelics used their knowledge to both provide benefits and impose costs on others. Shamans had ritual and medicinal functions relying on special knowledge and supernatural qualities that generated fear (e.g., an alleged capacity to magically kill others). Thus, according to Garfield et al. (2020) shamanism appears to be a distinct form of leadership that combines a strategy of inducing fear, similar to the dominance strategy, but is based on knowledge and expertise, similar to the prestige strategy. Shamans thus attained influential positions of leadership through their charisma and knowledgeability, social unification, healing competence, and use of supernatural powers to cause harm (Winkelman, 2010, 2021a). Artistic performance (often involving displays that incorporate superhuman abilities) is also implicated in this style of leadership (Singh, 2017).

How do we know that psychedelics were not frequently used by shamans in a Machiavellian way to mislead other community members to their own advantage (and to the detriment of their followers)? The anthropological record shows that while there are some shamans who have abused their positions, there are also those who do not act solely for personal gains, but who go about their functions without regard for material or political considerations (Ripinsky-Naxon, 1993). For instance, according to Sieroszewski (1900), who spent over a decade with the Yakuts (whose shamans employ the *Amanita muscaria* mushroom; a GABAergic hallucinogen), the “great” shamans were clearly distinct from the “middling” and the “mocking” or deceitful shamans, in the sense that they had not only material gain in view but the alleviation of the griefs of their fellow people, which was evident in the way they undertook their duties: with genuine conviction, negligence of personal danger, and inspired by the high ideal of sacrifice. Such shamans, he argued, always exerted an enormous influence over their audience.

Shamans are performers of the first order, enacting struggles with spiritual forces or magical flights to other realities, singing, dancing, and composing poetry (Estrada, 1989; Cardeña and Beard, 1996). Importantly, the shaman’s exhibition of power is carried out in service of the community, usually in public rituals (Langdon, 1992; Winkelman, 2021a) – the reactions of the audience enhancing the shamans’ prestige and efficacy (Cardeña and Beard, 1996). This public scrutiny and the ambiguous position of shamans in society (associated with the fact that they may use their power in negative ways, especially when directed against enemies outside of the social group) meant, as exemplified among the Aguaruna (whose shamans employ the psychedelic brew ayahuasca), that if results (e.g., of a healing session) are not forthcoming, the shaman himself may be suspected of, and punished for, sorcery (Brown, 1989). While shamans are known to engage in shamanic rivalries, wars, and duplicity (see e.g., Hugh-Jones, 1996), ethical training is a key element of the shaman’s education (Harner, 1980; Dow, 1986; Walsh, 1990; Krippner, 2002). Thus, shamans were dedicated to ending suffering, even it if required them to forgo their own comfort (see e.g., Dow, 1986), and had a personal interest in maintaining a good reputation (even if just for self-preserving reasons).

To be clear, shamanism includes a number of tricks of the trade such as sleight-of-hand, ventriloquism, surreptitiously using informants to obtain information about the patient, and the prototypical “sucking” cure (Eliade, 1972), which may be construed as involving deceit. However, these activities can be more properly considered as a form of “truthful trickery,” in the sense that they are procedures that engender a sense of mastery on the part of the client (e.g., specific techniques such as ventriloquism are used by shamans to indicate the presence of spirits) (Cardeña and Beard, 1996). Moreover, the use of acting and the employment of illusions (e.g., the use of masks and costumes) intentionally seek to affect the thoughts, feelings, or perceptions of the audience, bringing about emotional arousal and the evocation of faith, hope, and trust that ultimately enhance client expectations (Krippner, 2002). Hansen (2001) has compiled dozens of examples of shamanic trickery from the anthropological literature, adding that they may promote healing. As Cardeña and Beard (1996) argue, therapeutically speaking, pretense, role-playing, and performed illusions can go a long way in impressing onlookers, much more so than words alone. Undoubtedly, illusory cures can have concrete and real effects, as demonstrated by the placebo effect (Oken, 2008). The use of psychedelics to enhance suggestibility could have conferred a number of selective advantages in enhancing the ritual-induced placebo and hypnotic effects (see Rossano, 2007, 2009), as well as through inducing shared world views, and enhancing stress-reducing spiritual adaptations.

Therefore, while it may be concluded that shamans engage in deception and, perhaps, self-deception, as maintained by Warner (1980), a valid alternative perspective, as presented by Cardeña and Beard (1996: 33) is that shamans “give concrete form and shape to a vague, ungraspable disease, and that by this and other means the expectations of a possible cure are enhanced.” Shaman’s adroit maneuvering and use of legerdemain is thus justified by their cause: promoting individual and community health and well-being (Hansen, 2001). So, for instance, sleight-of-hand involving object extraction can be construed as the enactment of a spiritual struggle through which the shaman is able to remove a noxious influence on the patient’s welfare (Turner, 1964). Likewise, shamanic rituals in which psychedelics are consumed by the shaman and others to supposedly contact spirit worlds are an effective way to produce a community of experience, acting as a vector of affiliation to the social group and favoring the efficient transmission of metaphysical propositions relating to the supernatural realm (Dupuis, 2021).

It has been argued that human social evolution involved two key steps: first, early humans began to cooperate more and across wider interdependent networks; and second, humans became more group-minded, conforming to social norms in culturally marked groups and punishing norm-violators (Sterelny, 2007; Tomasello et al., 2012; Jensen et al., 2014). In this context of interdependence and mutualistic collaboration brought about by an obligate collaborative foraging lifeway, individuals had a direct interest in the well-being of their partners. This led to humans’ tendency to socially select others with regard to their cooperative behaviors, involving reputation-based social selection (including a concern for self-reputation as a cooperator) (Tomasello et al., 2012; Tomasello, 2014). In this context of joint intentionality and social selection against cheaters, it is hard to imagine that shamans using psychedelics to malignantly delude others could rise to positions of authority and succeed in maintaining power. Only shamans with exceptional abilities relative to others gain status (Garfield et al., 2020), and this involves providing benefits to the community as well as imposing reasonable costs (people accept this costs because they entail assurance of supernatural protection from a powerful figure). This capacity is directly related to their cognitive capital, involving medicinal and ritual knowledge. So, shamans that only imposed costs (e.g., threatening to harm others) without providing benefits (e.g., healing others) could not have gained status and would have likely been deemed cheaters deserving of shunning, ostracization, or even death. Concomitantly, the concern for self-reputation as a cooperator would have likely disincentivized free-riding or cheating strategies from psychedelic-using shamans since anyone with a poor reputation would have been avoided in the first place.

Moreover, an ability to mediate and resolve conflict (i.e., social unification capacities), healing abilities, and artistic prowess are hard-to-fake, costly behaviors that are interpreted as signaling commitment to the group’s well-being. Individuals using psychedelics, pretending to be able to provide benefits to the community (but in effect acting in the detriment of their followers) would have found it very difficult to fake these qualities convincingly, making deception by shamans much less likely. To gain prestige and maintain authority shamans needed to show charisma and ostensibly display their capacity to enter into contact with supernatural realms and powers, but they also were required to demonstrate to others their specialized knowledge by effectively healing and resolving social conflicts. In this way, the potential threat posed by fake, ineffective, or effectively damaging shamans was likely not much of an issue among the simple, smaller scale foraging societies that emerged over the course of the Pleistocene, given the scrutiny of followers (involving social selection based on reputation as a good collaborator); the special incentives for helping partners altruistically in a cooperative foraging context (which enhances the ability to suppress selfishness); and the difficulty of convincing others of having prosocial intentions without “tangible” or persuasive outcomes regarding healing and/or social unification.

### Psychedelic Instrumentalization as an Enabling Factor in the Construction of the Socio-Cognitive Niche

The evolutionary scenario put forward suggests that dietary incorporation of psilocybin, and its eventual integration into communal practice and proto-religious activity may have helped hominins respond adaptively to the socio-cognitive niche. Given that the socio-cognitive niche: (a) is simultaneously selection pressure and adaptive response (Downey and Lende, 2012); and (b) was partially constructed by hominins through their metabolism, their activities, and their choices (Laland et al., 2016), a second key aspect of this model is that psychedelic instrumentalization had niche-constructing effects that concomitantly aided in the creation and evolution of the socio-cognitive niche (right side of Figure 1). We hypothesize that the presence of psychedelics in the hominin social environment had significant consequences on the selective regime that drove hominin cognitive and behavioral evolution because it facilitated the construction of the adapted social environments that in turn selected for enhancements in the same underlying human capabilities that sustained the socio-cognitive niche. Interpersonal and prosocial effects of psilocybin would have mediated the expansion of social bonding mechanisms such as laughter, singing, dancing, storytelling, and religion, generating feedback and an ecological inheritance (see below for definitions of these concepts) that systematically biased the human evolutionary trajectory toward a socio-cognitive niche.

Niche construction is a process whereby organisms actively construct their environments and consequently change the conditions that effect selection (Odling-Smee et al., 2003). This implies that the products of an organism’s behavior are part of its selective environment. If organisms evolve in response to selection pressures modified by their ancestors, there is feedback in the system (Odling-Smee et al., 2003). So, for example, in the case of humans, fire use created selection for biological adaptations to cooked food (Wrangham, 2009); increased and diversified “tool” use favored neurobiological changes sustaining innovation and instruction–learning (Iriki and Taoka, 2012; Stout and Chaminade, 2012); and, more recently, dairy farming selected for lactase persistence (Tishkoff et al., 2007). The niche construction perspective thus recognizes human activities as directing human evolution (Odling-Smee et al., 2003; O’Brien and Laland, 2012). It also emphasizes the interactions between genetic and cultural processes over evolutionary time (Richerson et al., 2010). From this viewpoint, acquired characteristics and byproducts can become evolutionarily significant through evolutionary niches and ecological inheritance, which involves the passing on to descendants of inherited resources and conditions, and associated modified selection pressures (Laland et al., 2014, 2016)^2^.

Consequently, psychedelic instrumentalization can be modeled as an enabling factor in the hominization process, as a socially learned and culturally evolved trait (initially an individually learned self-medicative behavior) that assisted in the construction of the socio-cognitive niche. We argue this is the case because if early enhancements in the tendency to develop social links and cooperate in groups, in creativity, in non-verbal and linguistic expression, or suggestibility were actually produced by psychedelics (as suggested in the section on *Psychedelic instrumentalization in the human socio-cognitive niche*) they would have transformed the social environment, and thus the selection pressures, for ancient hominins. Psychedelic use could have sustained a feedback loop: it increased social cognition and symbolic behavior and thereby selected for yet further increases in such capacities by increasing the richness and complexity of the (constructed) social and semiotic environment.

In this way, the acquisition of enhanced cognition and sociality by members of the population that instrumentalized psychedelics would have intensified the selection pressures on members of descendant generations to develop visual representations, intelligence, and cooperation skills. Through a Baldwin effect [a non-Lamarckian way for environmentally induced somatic modifications, resulting from either learning or physiological adaptation, to become heritable changes (Jablonka and Lamb, 1998; Weber and Depew, 2003)], selection for genetic variants that make the acquisition of creativity and sociality faster, more reliable, and less dependent on environmental signals (such as the ritual consumption of psychedelics) would have eventually occurred.

Early humans learned by doing in an environment seeded with informational resources (indexical, iconic, and eventually symbolic) without explicit instruction and without formalized institutions (Sterelny, 2012, 2014). Our ancestors integrated these diverse semiotic elements in their collaborative efforts to interact with, and modify, their local physical and social ecologies, producing alterations in those ecologies that created new potentials for evolutionary dynamics (Fuentes, 2015). Semiotic elements, as well as artifacts and modified aspects of the landscape, contributed to a legacy of changed selection pressures (bequeathed generation through generation) that scaffolded the development and selection of adaptive traits. Thus, if psilocybin systematically increased the frequency of laughter, music-making (including dancing), ritualization, and prosocial leadership in ancient populations – thus enhancing the strength and quality of social bonds and decision-making, and consequently modifying the conditions for selection – then psilocybin could have ultimately influenced human evolution because such (ecologically and culturally inherited) communally held aspects of culture could exercise influences that selected in turn for genetic variants enhancing sociality, cognition, and communication skills.

As discussed above, construction of the socio-cognitive niche entailed expansion of a set of informal religious activities or “wild traditions” (*sensu* Boyer, 2019) involving leaders with supernatural qualities (shamans, in the broad sense; Garfield et al., 2020) that ritually induced altered states of consciousness (involving interactions with presumed superhuman powers and supernatural entities) to provide prosocial services of healing and divination (Winkelman, 2010, 2021c). These magico-religious activities were rooted in dancing, singing, and enactment that both induced alterations of consciousness and further served as a means for enhancing peace-making, affiliation, and imagination in community-wide nighttime healing and social effervescence rituals (Dunbar, 2017, 2020; Sterelny, 2018; Winkelman, 2021a). Psilocybin has a capacity to amplify these ecstatic and visionary thinking modalities through inducing ego-dissolution (Lebedev et al., 2015); a sense of connectedness (Carhart-Harris et al., 2018a); increased elementary and complex imagery (Kometer and Vollenweider, 2016); and entity-encounter occurrences (Lutkajtis, 2020). In an attempt to endure, make sense of, and communicate such intense, self-defining experiences humans often deploy rhythmic, hermeneutical, and rhetorical activity (Doyle, 2011, also see Munn, 1973 and Sterelny, 2018). Consequently, it is likely that repeated exposures to psilocybin mushrooms in ancestral human populations constituted an important influence on the origins and development of ancient religiosity, which comprised animism, belief in an afterlife, and shamanistic concepts (see Peoples et al., 2016 for a reconstruction of ancestral character states of religion; also Winkelman, 2010, 2013a, 2021b).

This means that early instantiations of animistic thinking and proto-shamanistic behavior prompted by psychedelic-induced altered states of consciousness (ASC) could have created contexts that effected subsequent selection enhancing human religiosity. If psychedelics engendered mental states that had adaptive effects on health, social bonding, and decision-making, this would have led to subsequent selection (both genetic and cultural) for the ability and motivation to alter consciousness through alternative (non-drug) means, and particularly in ways that most effectively functioned to promote salutogenesis, sociality, and creativity.

Accordingly, humans across the world and through time deployed various techniques to mimic, supplement, or amplify psychedelics’ effects, which involve stressing the cognitive system through sleep deprivation, temperature extremes, sensory overload, exhaustion, and emotionally charged, intense experience (Baumard and Boyer, 2013; Winkelman, 2013c). Ritual chanting, music, and dance were developed to induce euphoria and ecstasy (i.e., ASC) (Nettl, 1956; Winkelman, 1992, 2019b; Becker, 2004), enhancing health and well-being (Winkelman, 2008; MacDonald, 2013), social bonding (Savage et al., 2020), and creativity (Passanisi et al., 2015) even in the absence of psychedelic ingestion. Concomitantly, mythological narratives and religious beliefs were elaborated that enabled individual integration (McNamara, 2009) and collective accommodation (McKay, 2018; Sterelny, 2018) of the profound, and often unsettling, visionary experiences encountered in ASC. We think this niche-construction dynamic, which involves a combination of cultural and biological selection, helps explain why psychedelic use is not ubiquitous while institutionalized ASC (Bourguignon, 1973), music (Mehr et al., 2019), and religion (Norenzyan, 2010) are cross-cultural universals.

So, while psychedelic instrumentalization may have been an important ancient feature of human social and cognitive lives, it is now largely absent from most human cultures. Perhaps it suffered a fate similar to that of hunting and stone tool making (both also being mostly absent in modern human societies, having been replaced by agriculture and the use of metal tools) because safer, more effective and/or convenient (albeit perhaps less powerful) means to access ASC and support health, social bonding, and decision-making (e.g., music and religion) are now an integral part of the human niche.

## Conclusion and Future Directions

This article presents a model of adaptive utilization of psychedelics based on homeostatic and instrumentalization perspectives that explain potential selective advantages bestowed by psychedelics to hominins and archaic humans. Psilocybin ingestion could have provided homeostatic utility to our ancestors as a “treatment” for 5-HT depletion – a recurrent adaptive problem throughout advancement into a socio-cognitive niche. We show that, afterward, psychedelics could have increased adaptability and fitness in the context of this obligatorily cooperative, social-learning-dependent lifestyle because they could be harnessed as “instruments” to enhance performance of non-drug-related behaviors, particularly: to manage psychological distress and treat health problems; to improve social interaction and interpersonal relations; to facilitate collective ritual and religious activities; and to enhance group decision-making.

Niche-construction and gene-culture coevolutionary processes explain how dietary and societal incorporation of psychedelics may have become evolutionarily significant. The suggestion put forward is that psychedelics supported the elaboration of socially constructed environments – involving collective rituals, synchronic activities, and guidance by prosocial leaders – that could persist even if psychedelic instrumentalization was no longer an active part of the hominin behavioral repertoire. This means that psychedelic instrumentalization acted as an enabling factor in the development of the human socio-cognitive niche by mediating the expansion of ritual alterations of consciousness, healing, social bonding, and decision-making activities that, in turn, accelerated the rate at which key biological components of sociality, cognition, and communication skills spread in our lineage.

A topic worthy of further attention concerns psychedelics’ role in human brain evolution. Considered in the context of the changes in brain size and complexity that accompanied entry into the socio-cognitive niche, the ability of psychedelics to act as “psychoplastogens” (i.e., to rapidly promote induced synaptogenesis and neural plasticity: Ly et al., 2018) could have aided release of energetic constraints on encephalization, similar to how increased consumption of meat and energy-dense plant foods (e.g., fruit, tubers) was necessary for humans to overcome the metabolic constraints on brain expansion (Aiello and Wheeler, 1995; also see Milton, 2003). The presence of psychedelics in the early human diet may have also favored positive selection for exaggerated cortical plasticity – which apparently is a uniquely human derivation (Krubitzer, 2009) – and for the expansion of key functional networks involved in the enhancement of cognitive functions in humans compared to other primate species. Through their agonist action at 5-HT_2A_ receptors, psychedelics elevate synaptic efficacy and neuroplasticity (Ly et al., 2018) and functionally modulate the activity and connectivity of the frontoparietal network and the DMN (Carhart-Harris and Friston, 2019; Vollenweider and Preller, 2020), enhancing (potentially in the long-term) cognitive functioning (Cini et al., 2019) and sociality (Preller and Vollenweider, 2019). It has also been shown that psychedelic effects can reduce symptoms of autism (Sigafoos et al., 2007) and mimic certain aspects of psychosis (Carhart-Harris et al., 2016a). Intriguingly, the rapid evolutionary cortical expansion and reorganization in the human brain is most pronounced in higher-order cognitive networks (especially the frontoparietal network and DMN), and runs parallel (most pronouncedly in the DMN) with high expression of human-accelerated genes (HAR genes) involved in synapse and dendrite formation (Wei et al., 2019). Moreover, HAR and DMN genes show significant associations with individual variations in DMN functional activity, intelligence, social behavior, and mental conditions such as schizophrenia and autism (Wei et al., 2019).

An important matter that remains to be properly addressed concerns the question of why psychedelics affect our minds in the way they do. The homeostatic and drug instrumentalization perspective we have developed suggests that despite being chemical defenses designed to deter consumption (mainly by insects), psilocybin and other psychedelics were likely exploited by humans because of their chemical resemblance to endogenous signaling molecules that are fundamental but hard for the body to produce (in this case, 5-HT). Therefore, we surmise that the answer to the question of why psychedelics affect our minds in the way they do involves the coevolutionary chemical arms races between plants (fungi) and insects that selected for secondary metabolite synthesis of compounds that can similarly affect the nervous systems of insects and humans through common intercellular signaling pathways that plants, humans, and mushrooms share (Kennedy, 2014). This created the opportunity for what Schultz (2002) has referred to as “phylogenetic espionage” between plants, animals, and mushrooms, involving the capacity of animals to adaptively exploit potentially toxic fungal and vegetal secondary compounds. Moreover, animals typically engage in self-medicative and drug instrumentalization behaviors to solve adaptive problems directly related to their way of life (Forbey et al., 2009; Müller, 2020). Therefore, the answer to this question also resides in the specifics of the human lifeway, which placed a premium on social tolerance and cognitive flexibility (both regulated by 5-HT; Tricklebank and Daly, 2019) making the ingestion of 5-HT-mimicking psychedelics potentially beneficial and adaptive.

While observational evidence shows non-human mammals using plants for their psychoactivity (Siegel, 2005; Forbey et al., 2009) most animals are not particularly fond of mind-altering materials. Although self-medicative consumption of pharmacologically active plants occurs among animals (Huffman, 1997; Villalba and Provenza, 2007), only humans intentionally and periodically self-administer psychoactive compounds in the context of shared intentionality (i.e., coordinating their behavior and intentional states in cultural and conventional ways, e.g., in entrained ritual or dance) and manifest a propensity to employ non-pharmacological techniques to produce ASC (which likely indicates a derived preference for mind-alteration). Humans also possess an exceptional culturally accumulated knowledge about the toxic and intoxicating properties of plants, an ethnobotanical lore closely interwoven with mythology and under the purview of leaders with ritual/medicinal functions and supernatural qualities (Rätsch, 2005; Wink and van Wyk, 2008; Kennedy, 2014; Garfield et al., 2020). This shows that human ancestors – in a taxonomically distinctive way – constructed a niche that functionally and adaptively integrated certain mind-altering substances (i.e., hallucinogens, stimulants, and narcotics) into culture.

Importantly, archaic humans manifested derived traits such as advanced mindreading capacities, a propensity for shared rhythmical movement and sound, and early precursors of storytelling through mimesis (Sterelny, 2018; Dunbar, 2020). These are expected to have greatly enhanced the value of the “psychedelic experience” (Winkelman, 2021c) and its emotional, aesthetic, and hermeneutical appeal, thus explaining motivation for continued use of psychedelics in humans despite the fact that they do not have rewarding effects in animal models (see Heal et al., 2018). Although there is certain level of recreational abuse of psychedelics in humans (Murnane, 2018), psychedelics seem to have very little appeal to nonhuman primates and rodents and are considered to be a false negative of self-administration procedures (Calvey, 2019). Motivation to consume psilocybin is specifically related to visual effects, positive mood, insight, positive social effects, increased awareness of beauty (both visual and music), awe/amazement, meaningfulness, and mystical experience (Carbonaro et al., 2020). Hence, the nature of the reinforcement in humans seems to be intimately related to our enriched intersubjective, social, and symbolic life, and to the cognitive capacities that sustain that life, involving “perceptions of greater awareness, increased understanding, or profound insights that would have no counterpart in lower species with a less developed frontal cortex” (Nichols, 2004: 138).

In spite of this situation, it is unclear if psychedelic use was established relatively early in hominin life and thus may help explain the evolution of the socio-cognitive niche (as suggested here); or alternatively, if it emerged relatively late in human life (and was able to coincidentally or accidentally enhance cognition and sociality), in which case the focus should be instead on their coevolutionary interaction after independent origins. Evidence reviewed above concerning early hominins’ paleodiet and paleoecology, primate phylogeny of mycophagical and self-medicative behaviors, and the biogeography of psilocybin-containing fungi supports the former scenario. But, in all probability, exactly when our ancestors first deliberately employed consciousness-altering substances in rituals will forever remain uncertain. Nonetheless, more empirical research should enable us to properly evaluate the possible role and impact of the consumption of psychedelics in human adaptation and evolutionary history.

Moving forward, we think that research should seek to test some of the predictions that follow from the model, in an attempt to falsify them. For instance, the psychedelic instrumentalization model does not necessitate nor predict that psychedelic use is universal, or even widespread among cultures; but it does predict that psychedelic use should be more prevalent among foragers than agriculturalists (because it is assumed that psychedelic use is a relatively ancient behavior associated with simpler foraging societies and their shamanistic practices, which tend to disappear as agriculture intensifies; see Winkelman, 2021a). Systematic cross-cultural research methods could be used to test this prediction by empirically determining the frequency of psychedelic use as a function of subsistence type and political integration in a worldwide sample of societies that has been randomly selected through established probabilistic sampling procedures. Another *a priori* hypothesis that can be tested against the available cross-cultural evidence (e.g., from sources such as eHRAF) is that psychedelic use is mainly associated with specific functional contexts (i.e., healing, social bonding/socialization, and decision-making rituals). This notion is suggested by a qualitative reading of the anthropological record, but has yet to be confirmed by more systematic studies. Such cross-cultural similarities related to functionality, if verified, would suggest selection by biological and/or cultural evolution.

Moreover, the model predicts that psilocybin (and other serotonergic psychedelics) can substitute for 5-HT under conditions of tryptophan depletion, thereby ameliorating the costs associated with impairment of serotonergic neural signaling (involving, e.g., depressed mood, increased stress vulnerability, and cognitive inflexibility). This prediction can be subjected to a critical test by employing established experimental procedures for modifying peripheral and central 5-HT levels (that manipulate tryptophan levels, acutely or chronically, by depletion or supplementation; see Jenkins et al., 2016) in conjunction with the administration of different doses of psychedelic substances. For instance, it has been shown that tryptophan depletion produces significant reductions in the level of cooperation shown by participants in the context of a mixed-motive game, the Prisoner’s Dilemma (Wood et al., 2006). It is predicted that administration of psilocybin should revert the deficits in cooperation observed under such experimental conditions.

Obtaining negative results from these empirical tests would imply that the hypothetical scenario proposed here is unlikely.

Besides advocating for the relevance of an evolutionary perspective in the task of explaining and therapeutically harnessing the effects of psychedelics, it is hoped that this article will help encourage the incorporation of psychedelics into theoretical and empirical efforts directed at further advancing our understanding of the evolution of human behavior.

## Data Availability Statement

The original contributions presented in the study are included in the article/supplementary material, further inquiries can be directed to the corresponding author/s.

## Author Contributions

JR presented the idea of psychedelic instrumentalization in the socio-cognitive niche in a manuscript sent to MW. Both authors contributed to all aspects of the current manuscript.

## Conflict of Interest

The authors declare that the research was conducted in the absence of any commercial or financial relationships that could be construed as a potential conflict of interest.

## Publisher’s Note

All claims expressed in this article are solely those of the authors and do not necessarily represent those of their affiliated organizations, or those of the publisher, the editors and the reviewers. Any product that may be evaluated in this article, or claim that may be made by its manufacturer, is not guaranteed or endorsed by the publisher.

## Footnotes

1 We focus on psilocybin mushrooms as likely candidates for early psychedelic consumption in our lineage for the reasons enumerated here and because they require no preparation whatsoever, being bioactive in their natural state. Psychedelic plants in general were also ubiquitous and, in some instances, readily available (though certain plants required further processing to extract psychoactive secondary compounds) (Rätsch, 2005; Wink and van Wyk, 2008; Pennacchio et al., 2010; Alrashedy and Molina, 2016).

2 The niche construction perspective involves a triple-inheritance system comprised by genetic inheritance plus ecological inheritance and cultural inheritance (in humans and other cultural animals), which means that there are several relevant extra-genetic factors involved in evolutionary processes (for a diagrammatic explanation of this process see Figure 6.1 in Odling-Smee et al., 2003).

## References

[B1] Aiello L. C. Wheeler P. (1995). The expensive-tissue hypothesis. *Curr. Anthropol.* 36 199–221.

[B2] Akers B. P. Ruíz J. F. Piper A. Ruck C. A. P. (2011). A prehistoric mural in Spain depicting neurotropic Psilocybe mushrooms? *Econ. Bot.* 65 121–128. 10.1007/s12231-011-9152-5

[B3] Alrashedy N. A. Molina J. (2016). The ethnobotany of psychoactive plant use: a phylogenetic perspective. *PeerJ* 4:e2546. 10.7717/peerj.2546 27761334 PMC5068365

[B4] Antón S. C. Potts R. Aiello L. C. (2014). Evolution of early *Homo*: an integrated biological perspective. *Science* 345:1236828. 10.1126/science.1236828 24994657

[B5] Antón S. Snodgrass J. (2012). Origins and evolution of genus *Homo*: new perspectives. *Curr. Anthropol.* 53 S479–S496. 10.1086/667692

[B6] Baggott M. J. (2015). Psychedelics and creativity: a review of the quantitative literature. *PeerJ PrePrints* 3:e1202v1. 10.7287/peerj.preprints.1202v1

[B7] Barnouw V. (1950). Acculturation and Personality among the Wisconsin Chippewa. American Anthropological Association Memoir No. 72. *Am. Antiquity* 17(1Part1):75. 10.1017/S0002731600009094

[B8] Barrett F. S. Doss M. K. Sepeda N. D. Pekar J. J. Griffiths R. R. (2020). Emotions and brain function are altered up to one month after a single high dose of psilocybin. *Sci Rep* 10:2214. 10.1038/s41598-020-59282-y 32042038 PMC7010702

[B9] Barrett H. C. Cosmides L. Tooby J. (2007). “The hominid entry into the cognitive niche,” in *The Evolution Of Mind: Fundamental Questions And Controversies*, eds Gangestad S. W. Simpson J. A. (New York, NY: Guilford Press), 241–248.

[B10] Baumard N. Boyer P. (2013). Religious beliefs as reflective elaborations on intuitions: a modified dual-process model. *Curr. Direct. Psychol. Sci.* 22 295–300. 10.1177/0963721413478610

[B11] Becker J. (2004). *Deep Listeners: Music, Emotion, and Trancing.* Bloomington: Indiana University Press.

[B12] Benítez-Burraco A. Clay Z. Kempe V. (2020). Editorial: self-domestication and human evolution. *Front. Psychol.* 11:2007. 10.3389/fpsyg.2020.02007 32982840 PMC7477284

[B13] Berger M. Gray J. A. Roth B. L. (2009). The expanded biology of serotonin. *Annu. Rev. Med.* 60 355–366. 10.1146/annurev.med.60.042307.110802 19630576 PMC5864293

[B14] Bernhard H. Fehr E. Fischbacher U. (2006). Group affiliation and altruistic norm enforcement. *Am. Econ. Rev.* 96 217–221.

[B15] Bertolotti T. Magnani L. (2017). Theoretical considerations on cognitive niche construction. *Synthese* 194 4757–4779. 10.1007/s11229-016-1165-2

[B16] Boehm C. (1993). Egalitarian behavior and reverse dominance hierarchy. *Curr. Anthropol.* 34 227–254. 10.1086/204166

[B17] Bourguignon E. (1973). *Religion, Altered States of Consciousness, and Social Change.* Columbus, OH: Ohio State University Press.

[B18] Bouso J. dos Santos R. Alcazar-Corcoles M. Hallak J. (2018). Serotonergic psychedelics and personality: a systematic review of contemporary research. *Neurosci. Biobehav. Rev.* 87 118–132. 10.1016/j.neubiorev.2018.02.004 29452127

[B19] Boyd R. Richerson P. J. Henrich J. (2011). The cultural niche: why social learning is essential for human adaptation. *Proc. Natl. Acad. Sci. U.S.A.* 108 10918–10925. 10.1073/pnas.1100290108 21690340 PMC3131818

[B20] Boyer P. (2019). Informal religious activity outside hegemonic religions: Wild traditions and their relevance to evolutionary models. *Relig. Brain Behav.* 10 459–472. 10.1080/2153599X.2019.1678518

[B21] Boyer P. (2020). Why divination?: evolved psychology and strategic interaction in the production of truth. *Curr. Anthropol.* 61 100–123. 10.1086/706879

[B22] Boyer P. Liénard P. (2020). Ingredients of ‘rituals’ and their cognitive underpinnings. *Phil. Trans. R. Soc. B* 375:20190439. 10.1098/rstb.2019.0439 32594867 PMC7423267

[B23] Branchi I. (2011). The double-edged sword of neural plasticity: increasing serotonin levels leads to both greater vulnerability to depression and improved capacity to recover. *Psychoneuroendocrinology* 36 339–351. 10.1016/j.psyneuen.2010.08.011 20875703

[B24] Brouwer A. Carhart-Harris R. L. (2021). Pivotal mental states. *J. Psychopharmacol.* 35 319–352. 10.1177/0269881120959637 33174492 PMC8054165

[B25] Brown M. F. (1989). Dark side of the shaman. *Nat. Hist.* 11 8–10.

[B26] Bulbulia J. Geertz A. W. Atkinson Q. D. Cohen E. Evans N. Françoiset P. (2013). “The cultural evolution of religion,” in *Cultural Evolution: Society, Technology, Language, and Religion*, eds Richerson P. J. Christiansen M. H. (Cambridge, MA: MIT Press), 381–404.

[B27] Calvey T. (2019). Human self-domestication and the extended evolutionary synthesis of addiction: how humans evolved a unique vulnerability. *Neuroscience* 419 100–107. 10.1016/j.neuroscience.2019.09.013 31654715

[B28] Carbonaro T. M. Johnson M. W. Griffiths R. R. (2020). Subjective features of the psilocybin experience that may account for its self-administration by humans: a double-blind comparison of psilocybin and dextromethorphan. *Psychopharmacology (Berl).* 237 2293–2304. 10.1007/s00213-020-05533-9 32500212 PMC10013695

[B29] Cardeña E. Beard J. (1996). Truthful trickery: shamanism, acting and reality. *Perform. Res.* 1 31–39. 10.1080/13528165.1996.10871509

[B30] Carhart-Harris R. L. Friston K. J. (2019). REBUS and the anarchic brain: toward a unified model of the brain action of psychedelics. *Pharmacol. Rev.* 71 316–344. 10.1124/pr.118.017160 31221820 PMC6588209

[B31] Carhart-Harris R. L. Nutt D. (2017). Serotonin and brain function: a tale of two receptors. *J. Psychopharmacol.* 31 1091–1120. 10.1177/0269881117725915 28858536 PMC5606297

[B32] Carhart-Harris R. L. Roseman L. Haijen E. Erritzoe D. Watts R. Branchi I. (2018b). Psychedelics and the essential importance of context. *J. Psychopharmacol.* 32 725–731. 10.1177/0269881118754710 29446697

[B33] Carhart-Harris R. L. Erritzoe D. Williams T. Stone J. M. Reed L. J. Colasanti A. (2012). Neural correlates of the psychedelic state as determined by fMRI studies with psilocybin. *Proc. Natl. Acad. Sci. U.S.A.* 109 2138–2143. 10.1073/pnas.1119598109 22308440 PMC3277566

[B34] Carhart-Harris R. L. Giribaldi B. Watts R. Baker-Jones M. Murphy-Beiner A. Murphy R. (2021). Trial of psilocybin versus escitalopram for depression. *N. Engl. J. Med.* 384 1402–1411. 10.1056/NEJMoa2032994 33852780

[B35] Carhart-Harris R. L. Kaelen M. Bolstridge M. Williams T. M. Williams L. T. Underwood R. (2016a). The paradoxical psychological effects of lysergic acid diethylamide (LSD). *Psychol. Med.* 46 1379–1390. 10.1017/S0033291715002901 26847689

[B36] Carhart-Harris R. L. Kaelen M. Whalley M. G. Bolstridge M. Feilding A. Nutt D. J. (2015). LSD enhances suggestibility in healthy volunteers. *Psychopharmacology (Berl)* 232 785–794. 10.1007/s00213-014-3714-z 25242255

[B37] Carhart-Harris R. L. Leech R. Hellyer P. Shanahan M. Feilding A. Tagliazucchi E. (2014). The entropic brain: a theory of conscious states informed by neuroimaging research with psychedelic drugs. *Front. Hum. Neurosci.* 8:20. 10.3389/fnhum.2014.00020 24550805 PMC3909994

[B38] Carhart-Harris R. L. Muthukumaraswamy S. Roseman L. Kaelen M. Droog W. Murphy K. (2016b). Neural correlates of the LSD experience revealed by multimodal neuroimaging. *Proc. Natl. Acad. Sci. U.S.A.* 113 4853–4858. 10.1073/pnas.1518377113 27071089 PMC4855588

[B39] Carhart-Harris R. L. Erritzoe D. Haijen E. Kaelen M. Watts R. (2018a). Psychedelics and connectedness. *Psychopharmacology (Berl).* 235 547–550. 10.1007/s00213-017-4701-y 28795211

[B40] Carhart-Harris R. Goodwin G. M. (2017). The therapeutic potential of psychedelic drugs: past, present, and future. *Neuropsychopharmacology* 42 2105–2113. 10.1038/npp.2017.84 28443617 PMC5603818

[B41] Charles S. J. van Mulukom V. Farias M. Brown J. Delmonte R. Maraldi E. (2020). Religious rituals increase social bonding and pain threshold. *PsyArXiv* [Preprint] 10.31234/osf.io/my4hs 42336380

[B42] Cini F. A. Ornelas I. Marcos E. Goto-Silva L. Nascimento J. Ruschi S. (2019). d-Lysergic acid diethylamide has major potential as a cognitive enhancer. *bioRxiv* [Preprint] 10.1101/866814

[B43] Cosmides L. Tooby J. (2001). “Unravelling the enigma of human intelligence: evolutionary psychology and the multimodular mind,” in *The Evolution of Intelligence*, eds Sternberg R. J. Kaufman J. C. (New Jersey, NJ: Erlbaum), 145–198.

[B44] Curry P. (2010). “Embodiment, alterity and agency: Negotiating antinomies in divination,” in *Divination: Perspectives for a New Millennium*, ed. Curry P. (Abingdon: Ashgate), 85–118.

[B45] Davis A. K. Clifton J. M. Weaver E. G. Hurwitz E. S. Johnson M. W. Griffiths R. R. (2020). Survey of entity encounter experiences occasioned by inhaled N,N-dimethyltryptamine: phenomenology, interpretation, and enduring effects. *J. Psychopharmacol.* 34 1008–1020. 10.1177/0269881120916143 32345112

[B46] de Araujo D. B. Ribeiro S. Cecchi G. A. Carvalho F. M. Sanchez T. A. Pinto J. P. (2012). Seeing with the eyes shut: neural basis of enhanced imagery following ayahuasca ingestion. *Hum. Brain Mapp.* 33 2550–2560. 10.1002/hbm.21381 21922603 PMC6870240

[B47] De Gregorio D. Aguilar-Valles A. Preller K. H. Heifets B. D. Hibicke M. Mitchell J. (2021a). Hallucinogens in mental health: preclinical and clinical studies on LSD, psilocybin, MDMA, and ketamine. *J. Neurosci.* 41 891–900. 10.1523/JNEUROSCI.1659-20.2020 33257322 PMC7880300

[B48] De Gregorio D. Enns J. P. Nuñez N. A. Posa L. Gobbi G. (2018). d-Lysergic acid diethylamide, psilocybin, and other classic hallucinogens: mechanism of action and potential therapeutic applications in mood disorders. *Prog. Brain Res.* 242 69–96. 10.1016/bs.pbr.2018.07.008 30471683

[B49] De Gregorio D. Popic J. Enns J. P. Inserra A. Skalecka A. Markopoulos A. (2021b). Lysergic acid diethylamide (LSD) promotes social behavior through mTORC1 in the excitatory neurotransmission. *Proc. Natl. Acad. Sci. U.S.A.* 118 e2020705118. 10.1073/pnas.2020705118 33495318 PMC7865169

[B50] Dijour E. (1933). Les ceremonies d’expulsions des maladies chez les Matako. *J. Soc. Am.* 25 211–217.

[B51] Dobkin de Ríos M. (1984). *Hallucinogens: Cross-Cultural Perspectives.* Albuquerque: University of New Mexico Press.

[B52] Dolder P. C. Schmid Y. Müller F. Borgwardt S. Liechti M. E. (2016). LSD acutely impairs fear recognition and enhances emotional empathy and sociality. *Neuropsychopharmacology* 41 2638–2646. 10.1038/npp.2016.82 27249781 PMC5026740

[B53] Dole G. (1974). “The marriages of Pacho: a woman’s life among the Amahuaca,” in *Many Sisters: Women in Cross-Cultural Perspective*, ed. Mathiasson C. J. (New York, NY: Free Press), 3–35.

[B54] Domínguez-Rodrigo M. Pickering T. (2003). Early hominid hunting and scavenging: a zooarcheological review. *Evol. Anthropol.* 12 275–282. 10.1002/evan.10119

[B55] Domnauer C. (2020). The legume pod motif as a symbolic representation of the shamanic hallucinogen, Vilca (*Anadenanthera* spp.), in Pre-Columbian Andean cultures. *Ñawpa Pacha* 40 163–173. 10.1080/00776297.2020.1820654

[B56] dos Santos R. G. Osório F. L. Crippa J. A. S. Riba J. Zuardi A. W. Hallak J. E. C. (2016). Antidepressive, anxiolytic, and antiaddictive effects of ayahuasca, psilocybin and lysergic acid diethylamide (LSD): a systematic review of clinical trials published in the last 25 years. *Ther. Adv. Psychopharmacol.* 6 193–213. 10.1177/2045125316638008 27354908 PMC4910400

[B57] Dow J. (1986). *The Shaman’s Touch: Otomi Indian Symbolic Healing.* Salt Lake, UT: University of Utah Press.

[B58] Downey G. Lende D. H. (2012). “Evolution and the brain,” in *The Encultured Brain: An Introduction to Neuroanthropology*, eds Lende D. H. Downey G. (Cambridge, MA: MIT Press), 103–138.

[B59] Doyle R. M. (2011). *Darwin’s Pharmacy: Sex, Plants, and the Evolution of the Noösphere.* Seattle, WA: University of Washington Press.

[B60] Dunbar R. I. M. (2010). The social role of touch in humans and primates: behavioural function and neurobiological mechanisms. *Neurosci. Biobehav. Rev.* 34 260–268. 10.1016/j.neubiorev.2008.07.001 18662717

[B61] Dunbar R. I. M. (2014). *Human Evolution: A Pelican Introduction.* London: Pelican Books.

[B62] Dunbar R. I. M. (2017). What’s missing from the scientific study of religion? *Relig. Brain Behav.* 7 349–353. 10.1080/2153599X.2016.1249927

[B63] Dunbar R. I. M. (2020). Religion, the social brain and the mystical stance. *Arch. Psychol. Relig.* 42 46–62. 10.1177/0084672419900547

[B64] Dunbar R. I. M. Teasdale B. Thompson J. Budelmann F. Duncan S. van Emde Boas E. (2016). Emotional arousal when watching drama increases pain threshold and social bonding. *R. Soc. Open Sci.* 3:160288. 10.1098/rsos.160288 27703694 PMC5043313

[B65] Dupuis D. (2021). The socialization of hallucinations: cultural priors, social interactions, and contextual factors in the use of psychedelics. *Transcult. Psychiatry* 14:115. 10.1177/13634615211036388 PMC9660275 36367797

[B66] Durkheim E. (1995). *The Elementary Forms of Religious Life. (K. E. Fields, Trans.).* New York, NY: Free Press.

[B67] Eliade M. (1972). *Shamanism: Archaic Techniques Of Ecstasy (W. R. Trask, Trans.).* Princeton, NJ: Princeton University Press.

[B68] Erritzoe D. Roseman L. Nour M. MacLean K. Kaelen M. Nutt D. (2018). Effects of psilocybin therapy on personality structure. *Acta Psychiatrica Scand.* 138 368–378. 10.1111/acps.12904 29923178 PMC6220878

[B69] Espírito D. (2019). “Divination,” in *The Cambridge Encyclopedia Of Anthropology*, eds Stein F. Lazar S. Candea M. Diemberger H. Robbins J. Sanchez A. (Cambridge: University of Cambridge), 10.29164/19divination

[B70] Estrada A. (1989). *Vida de María Sabina: La sabia de los hongos.* Mexico: Siglo XXI.

[B71] Family N. Vinson D. Vigliocco G. Kaelen M. Bolstridge M. Nutt D. J. (2016). Semantic activation in LSD: evidence from picture naming. *Lang. Cogn. Neurosci.* 31 1320–1327. 10.1080/23273798.2016.1217030

[B72] Fehr E. Bernhard H. Rockenbach B. (2008). Egalitarianism in young children. *Nature* 454 1079–1083. 10.1038/nature07155 18756249

[B73] Ferreira Júnior W. S. Cruz M. P. Vieira F. J. Albuquerque U. P. (2015). “An evolutionary perspective on the use of hallucinogens,” in *Evolutionary Ethnobiology*, eds De Medeiros P. Casas A. (Cham: Springer), 185–197. 10.1007/978-3-319-19917-7_14

[B74] Fingelkurts A. A. Fingelkurts A. A. Kallio-Tamminen T. (2020). Selfhood triumvirate: from phenomenology to brain activity and back again. *Conscious Cogn.* 86:103031. 10.1016/j.concog.2020.103031 33099083

[B75] Fitzpatrick S. (Ed.) (2018). *Ancient Psychoactive Substances.* Gainesville, FL: University Press of Florida.

[B76] Flattery D. S. Schwartz M. (1989). *Haoma and Harmaline.* Berkeley, CA: University of California.

[B77] Forbey J. S. Harvey A. L. Huffman M. A. Provenza F. D. Sullivan R. Tasdemir D. (2009). Exploitation of secondary metabolites by animals: a response to homeostatic challenges. *Integr. Comp. Biol.* 49 314–328. 10.1093/icb/icp046 21665822

[B78] Fox K. C. R. Girn M. Parro C. C. Christoff K. (2018). “Functional neuroimaging of psychedelic experience: an overview of psychological and neural effects and their relevance to research on creativity, daydreaming, and dreaming,” in *The Cambridge Handbook Of The Neuroscience Of Creativity*, eds Jung R. E. Vartanian O. (Cambridge: Cambridge University Press), 92–113. 10.1017/9781316556238.007

[B79] Friedman M. (2018). Analysis, nutrition, and health benefits of tryptophan. *Int. J. Tryptophan Res.* 11 1–12. 10.1177/1178646918802282 30275700 PMC6158605

[B80] Froese T. Guzmán G. Guzmán-Dávalos L. (2016). On the origin of the genus Psilocybe and its potential ritual use in ancient Africa and Europe. *Econ. Bot.* 70 103–114. 10.1007/s12231-016-9342-2

[B81] Frost M. (2017). *Herbs That Madden, Herbs That Cure: A History Of Hallucinogenic Plant Use in Colonial Mexico.* Doctoral Dissertation. Charlottesville, VA: University of Virginia, 10.18130/V3QW96

[B82] Fuentes A. (2015). Integrative anthropology and the human niche: toward a contemporary approach to human evolution. *Am. Anthropol.* 117 302–315. 10.1111/aman.12248

[B83] Furst P. T. (Eds.) (1990). *Flesh of the Gods: The Ritual Use Of Hallucinogens.* Springfield, IL: Waveland.

[B84] Gabay A. S. Carhart-Harris R. L. Mazibuko N. Kempton M. J. Morrison P. D. Nutt D. J. (2018). Psilocybin and MDMA reduce costly punishment in the Ultimatum Game. *Sci. Rep.* 8:8236. 10.1038/s41598-018-26656-2 29844496 PMC5974271

[B85] Gable R. S. (2004). Comparison of acute lethal toxicity of commonly abused psychoactive substances. *Addiction* 99 686–696. 10.1111/j.1360-0443.2004.00744.x 15139867

[B86] Gallimore A. R. (2015). Restructuring consciousness–the psychedelic state in light of integrated information theory. *Front. Hum. Neurosci.* 9:346. 10.3389/fnhum.2015.00346 26124719 PMC4464176

[B87] Gamble C. Gowlett J. Dunbar R. (2014). *Thinking Big: How the Evolution Of Social Life Shaped the Human Mind.* New York, NY: Thames & Hudson.

[B88] Garcia-Romeu A. Kersgaard B. Addy P. H. (2016). Clinical applications of hallucinogens: a review. *Exp. Clin. Psychopharmacol.* 24 229–268. 10.1037/pha0000084 27454674 PMC5001686

[B89] Garfield Z. H. Syme K. L. Hagen E. H. (2020). Universal and variable leadership dimensions across human societies. *Evol. Hum. Behav.* 41 397–414. 10.1016/j.evolhumbehav.2020.07.012

[B90] Gintis H. van Schaik C. Boehm C. (2015). Zoon politikon: the evolutionary origins of human political systems. *Curr. Anthropol.* 56 327–353. 10.1086/681217 29581024

[B91] Girn M. Mills C. Roseman L. Carhart-Harris R. L. Christoff K. (2020). Updating the dynamic framework of thought: Creativity and psychedelics. *Neuroimage* 213:116726. 10.1016/j.neuroimage.2020.116726 32160951

[B92] Goldberg S. B. Pace B. Nicholas C. Raisone C. Hutson P. (2020a). The experimental effects of psilocybin on symptoms of anxiety and depression: a meta-analysis. *Psychiatry Res.* 284:112749. 10.1016/j.psychres.2020.112749 31931272

[B93] Goldberg S. B. Shechet B. Nicholas C. R. Ng C. W. Deole G. Chen Z. (2020b). Post-acute psychological effects of classical serotonergic psychedelics: a systematic review and meta-analysis. *Psychol. Med.* 50 2655–2666. 10.1017/S003329172000389X 33143790 PMC7855004

[B94] Griffiths R. R. Hurwitz E. S. Davis A. K. Johnson M. W. Jesse R. (2019). Survey of subjective “God encounter experiences”: comparisons among naturally occurring experiences and those occasioned by the classic psychedelics psilocybin, LSD, ayahuasca, or DMT. *PLoS One* 14:e0214377. 10.1371/journal.pone.0214377 31013281 PMC6478303

[B95] Griffiths R. R. Johnson M. W. Richards W. A. Richards B. R. McCann U. D. Jesse R. (2011). Psilocybin-occasioned mystical-type experiences: immediate and persisting dose-related effects. *Psychopharmacology (Berl)* 218 649–665. 10.1007/s00213-011-2358-5 21674151 PMC3308357

[B96] Griffiths R. R. Johnson M. W. Richards W. R. Richards B. D. Jesse R. MacLean K. A. (2018). Psilocybin-occasioned mystical-type experience in combination with meditation and other spiritual practices produce enduring positive changes in trait measures of prosocial attitudes and behaviors. *J. Psychopharmacol.* 32 49–69. 10.1177/0269881117731279 29020861 PMC5772431

[B97] Griffiths R. Richards W. McCann U. Jesse R. (2006). Psilocybin can occasion mystical-type experiences having substantial and sustained personal meaning and spiritual significance. *Psychopharmacology* 187 268–283. 10.1007/s00213-006-0457-5 16826400

[B98] Guerra-Doce E. (2014). The origins of inebriation: archaeological evidence of the consumption of fermented beverages and drugs in prehistoric Eurasia. *J. Archaeol. Method Theory* 22 751–782. 10.1007/s10816-014-9205-z

[B99] Guerra-Doce E. (2015). Psychoactive substances in prehistoric times: examining the archaeological evidence. *Time Mind* 8 91–112. 10.1080/1751696X.2014.993244

[B100] Guzmán G. (2005). Species diversity of the genus Psilocybe (Basidiomycotina, Agaricales, Strophariaceae) in the world mycobiota, with special attention to hallucinogenic properties. *Int. J. Med. Mushrooms* 7 305–332. 10.1615/intjmedmushr.v7.i12.280

[B101] Guzmán G. Allen J. Gartz J. (1998). A worldwide geographical distribution of the neurotropic fungi, an analysis and discussion. *Ann. Museo Civico Rovereto* 14 189–280.

[B102] Guzmán G. Nixon S. C. Ramírez-Guillén F. Cortés-Pérez A. (2014). Psilocybe s. str. (Agaricales, Strophariaceae) in Africa with description of a new species from the Congo. *Sydowia* 66 43–53.

[B103] Haden M. Woods B. (2020). LSD overdoses: three case reports. *J. Stud. Alcohol. Drugs* 81 115–118. 10.15288/jsad.2020.81.115 32048609

[B104] Hagen E. Roulette C. Sullivan R. (2013). Explaining human recreational use of ‘pesticides’: the neurotoxin regulation model of substance use vs. the hijack model and implications for age and sex differences in drug consumption. *Front. Psychiatry* 4:142. 10.3389/fpsyt.2013.00142 24204348 PMC3817850

[B105] Haijen E. Kaelen M. Roseman L. Timmermann C. Kettner H. Russ S. (2018). Predicting responses to psychedelics: a prospective study. *Front. Pharmacol.* 9:897. 10.3389/fphar.2018.00897 30450045 PMC6225734

[B106] Hansen G. P. (2001). *The trickster and the paranormal.* New York, NY: Xlibris.

[B107] Hanson A. Hodge K. Porter L. (2003). Mycophagy among primates. *Mycologist* 17 6–10. 10.1017/S0269915X0300106X

[B108] Hardy K. Buckley S. Huffman M. (2013). Neanderthal self-medication in context. *Antiquity* 87 873–878. 10.1017/S0003598X00049528

[B109] Hare B. (2017). Survival of the friendliest: *Homo sapiens* evolved via selection for prosociality. *Ann. Rev. Psychol.* 68 155–186. 10.1146/annurev-psych-010416-044201 27732802

[B110] Hare B. Tomasello M. (2005). The emotional reactivity hypothesis and cognitive evolution: reply to Miklósi and Topál. *Trends Cogn. Sci.* 9 464–465. 10.1016/j.tics.2005.08.010

[B111] Harner M. (1980). *The Way of the Shaman: A Guide to Power and Healing.* San Francisco, CA: Harper & Row.

[B112] Harner M. J. (1973). *Hallucinogens and Shamanism.* New York, NY: Oxford U. Press.

[B113] Hartogsohn I. (2016). Set and setting, psychedelics and the placebo response: an extra-pharmacological perspective on psychopharmacology. *J. Psychopharmacol.* 30 1259–1267. 10.1177/0269881116677852 27852960

[B114] Hartogsohn I. (2018). The meaning-enhancing properties of psychedelics and their mediator role in psychedelic therapy, spirituality, and creativity. *Front. Neurosci.* 12:129. 10.3389/fnins.2018.00129 29559884 PMC5845636

[B115] Heal D. J. Gosden J. Smith S. L. (2018). Evaluating the abuse potential of psychedelic drugs as part of the safety pharmacology assessment for medical use in humans. *Neuropharmacology* 142 89–115. 10.1016/j.neuropharm.2018.01.049 29427652

[B116] Heyes C. M. Frith C. D. (2014). The cultural evolution of mind reading. *Science* 344:6190. 10.1126/science.1243091 24948740

[B117] Horváth L. Szummer C. Szabo A. (2017). Weak phantasy and visionary phantasy: the phenomenological significance of altered states of consciousness. *Phenomenol. Cogn. Sci.* 17 117–129. 10.1007/s11097-016-9497-4

[B118] Hublin J. J. Richards M. P. (Eds.) (2009). *The Evolution of Hominin Diets: Integrating Approaches to the Study of Paleolithic Subsistence.* Berlin: Springer.

[B119] Huffman M. A. (1997). Current evidence for self-medication in primates: a multidisciplinary perspective. *Yearbook. Phys. Anthropol.* 40 171–200.

[B120] Hugh-Jones S. (1979). *The Palm and the Pleiades: Initiation and Cosmology in Northwest Amazonia.* New York, NY: Cambridge University Press.

[B121] Hugh-Jones S. (1996). “Shamans, prophets, priests and pastors,” in *Shamanism, History, and the State*, eds Thomas N. Humphrey C. (Ann Arbor: University of Michigan Press), 32–75.

[B122] Inserra A. De Gregório D. Gobbi G. (2021). Psychedelics in psychiatry: neuroplastic, immunomodulatory, and neurotransmitter mechanisms. *Pharmacol. Rev.* 73 202–277. 10.1124/pharmrev.120.000056 33328244

[B123] Iriki A. Taoka M. (2012). Triadic (ecological, neural, cognitive) niche construction: a scenario of human brain evolution extrapolating tool use and language from the control of reaching actions. *Phil. Trans. R. Soc. B* 367 10–23. 10.1098/rstb.2011.0190 22106423 PMC3223791

[B124] Jablonka E. Lamb M. J. (1998). Epigenetic inheritance in evolution. *J. Evol. Biol.* 11 159–183. 10.1046/j.1420-9101.1998.11020159.x

[B125] Jackson J. E. (1983). *The Fish People: Linguistic Exogamy and Tukanoan Identity in Northwest Amazonia.* New York, NY: Cambridge University Press, 10.1017/cbo9780511621901

[B126] Jenkins T. A. Nguyen J. C. Polglaze K. E. Bertrand P. P. (2016). Influence of tryptophan and serotonin on mood and cognition with a possible role of the gut-brain axis. *Nutrients* 8:56. 10.3390/nu8010056 26805875 PMC4728667

[B127] Jensen K. Vaish A. Schmidt M. F. H. (2014). The emergence of human prosociality: aligning with others through feelings, concerns, and norms. *Front. Psychol.* 5:822. 10.3389/fpsyg.2014.00822 25120521 PMC4114263

[B128] Johansen P. Ø Krebs T. S. (2015). Psychedelics not linked to mental health problems or suicidal behavior: a population study. *J. Psychopharmacol.* 29 270–279. 10.1177/0269881114568039 25744618

[B129] Johns T. (1990). *With Bitter Herbs They Shall Eat it: Chemical Ecology and the Origins of Human Diet and Medicine.* Tucson: The University of Arizona Press.

[B130] Johnson M. Griffiths R. (2017). Potential therapeutic effects of psilocybin. *Neurotherapeutics* 14 734–740. 10.1007/s13311-017-0542-y 28585222 PMC5509636

[B131] Johnson M. Richards W. Griffiths R. (2008). Human hallucinogen research: guidelines for safety. *J. Psychopharmacol.* 22 603–620. 10.1177/0269881108093587 18593734 PMC3056407

[B132] Kaelen M. Giribaldi B. Raine J. Evans L. Timmerman C. Rodriguez N. (2018). The hidden therapist: evidence for a central role of music in psychedelic therapy. *Psychopharmacology* 235 505–519. 10.1007/s00213-017-4820-5 29396616 PMC5893695

[B133] Kanen J. W. Apergis-Schoute A. M. Yellowlees R. Arntz F. E. van der Flier F. E. Price A. (2020). Serotonin depletion impairs both Pavlovian and instrumental reversal learning in healthy humans. *bioRxiv* [Preprint] 10.1101/2020.04.26.062463 PMC8873011 34429517

[B134] Kaplan H. Hill K. Lancaster J. Hurtado A. M. (2000). A theory of human life history evolution: diet, intelligence, and longevity. *Evol. Anthropol.* 9 156–185.

[B135] Katz R. (1984). *Boiling Energy: Community Healing Among the Kalahari Kung.* Cambridge: Harvard University. Press.

[B136] Kennedy D. O. (2014). *Plants and the Human Brain.* New York NY: Oxford University Press.

[B137] Kettner H. Rosas F. E. Timmermann C. Kärtner L. Carhart-Harris R. L. Roseman L. (2021). Psychedelic communitas: intersubjective experience during psychedelic group sessions predicts enduring changes in psychological wellbeing and social connectedness. *Front. Pharmacol.* 12:623985. 10.3389/fphar.2021.623985 33995022 PMC8114773

[B138] Klein R. G. (1999). *The Human Career: Human Biological and Cultural Origins.* Chicago, IL: University of Chicago Press.

[B139] Kohrt B. A. Ottman K. Panter-Brick C. Konner M. Patel V. (2020). Why we heal: The evolution of psychological healing and implications for global mental health. *Clin. Psychol. Rev.* 82:101920. 10.1016/j.cpr.2020.101920 33126037

[B140] Kometer M. Vollenweider F. X. (2016). “Serotonergic hallucinogen-induced visual perceptual alterations,” in *Behavioral Neurobiology of Psychedelic Drugs. Current Topics in Behavioral Neurosciences*, Vol. 36 eds Halberstadt L. Vollenweider F. X. Nichols D. E. (Berlin: Springer), 257–282. 10.1007/7854_2016_461 27900674

[B141] Kometer M. Pokorny T. Seifritz E. Volleinweider F. (2015). Psilocybin-induced spiritual experiences and insightfulness are associated with synchronization of neuronal oscillations. *Psychopharmacology (Berl)* 232 3663–3676. 10.1007/s00213-015-4026-7 26231498

[B142] Kometer M. Schmidt A. Bachmann R. Studerus E. Seifritz E. Vollenweider F. X. (2012). Psilocybin biases facial recognition, goal-directed behavior, and mood state toward positive relative to negative emotions through different serotonergic subreceptors. *Biol. Psychiatry* 72 898–906. 10.1016/j.biopsych.2012.04.005 22578254

[B143] Kometer M. Schmidt A. Jäncke L. Vollenweider F. X. (2013). Activation of serotonin2A receptors underlies the psilocybin-induced effects on α oscillations, N170 visual-evoked potentials, and visual hallucinations. *J. Neurosci.* 33 10544–10551. 10.1523/JNEUROSCI.3007-12.2013 23785166 PMC6618596

[B144] Kraehenmann R. Pokorny D. Aicher H. Preller K. H. Pokorny T. Bosch O. G. (2017). LSD increases primary process thinking via serotonin2A receptor activation. *Front. Pharmacol.* 8:814. 10.3389/fphar.2017.00814 29167644 PMC5682333

[B145] Kraehenmann R. Schmidt A. Friston K. Preller K. H. Seifritz E. Vollenweider F. X. (2016). The mixed serotonin receptor agonist psilocybin reduces threat-induced modulation of amygdala connectivity. *Neuroimage Clin.* 22 53–60. 10.1016/j.nicl.2015.08.009 26909323 PMC4732191

[B146] Krippner S. C. (2002). Conflicting perspectives on shamans and shamanism: points and counterpoints. *Am. Psychol.* 57 962–978. 10.1037/0003-066x.57.11.962 12564209

[B147] Krubitzer L. (2009). In search of a unifying theory of complex brain evolution. *Ann. N. Y. Acad. Sci.* 1156 44–67. 10.1111/j.1749-6632.2009.04421.x 19338502 PMC2666944

[B148] Kuypers K. P. C. (2019). Psychedelic medicine: the biology underlying the persisting psychedelic effects. *Med. Hypotheses* 125 21–24. 10.1016/j.mehy.2019.02.029 30902145

[B149] Kuypers K. P. Riba J. de la Fuente Revenga M. Barker S. Theunissen E. L. Ramaekers J. G. (2016). Ayahuasca enhances creative divergent thinking while decreasing conventional convergent thinking. *Psychopharmacology (Berl)* 233 3395–3403. 10.1007/s00213-016-4377-8 27435062 PMC4989012

[B150] Laland K. N. O’Brien M. J. (2011). Cultural niche construction: an introduction. *Biol. Theory* 6 191–202. 10.1007/s13752-012-0026-6

[B151] Laland K. Matthews B. Feldman M. W. (2016). An introduction to niche construction theory. *Evol. Ecol.* 30 191–202. 10.1007/s10682-016-9821-z 27429507 PMC4922671

[B152] Laland K. Uller T. Feldman M. Sterelny K. Müller G. B. Moczek A. (2014). Does evolutionary theory need a rethink? *Nature* 514 161–164. 10.1038/514161a 25297418

[B153] Langdon E. J. M. (1992). “Introduction: shamanism and anthropology,” in *Portals of Power: Shamanism in South America*, eds Langdon E. J. M. Baer G. (Albuquerque: University of New Mexico Press), 1–21.

[B154] Lebedev A. Lövdén M. Rosenthal G. Feilding A. Nutt D. Carhart-Harris R. (2015). Finding the self by losing the self: neural correlates of ego-dissolution under psilocybin. *Hum. Brain Mapp.* 36 3137–3153. 10.1002/hbm.22833 26010878 PMC6869189

[B155] Legare C. H. Nielsen M. (2020). Ritual explained: interdisciplinary answers to Tinbergen’s four questions. *Phil. Trans. R. Soc. Lond. B Biol. Sci.* 375:20190419. 10.1098/rstb.2019.0419 32594869 PMC7423255

[B156] Leptourgos P. Fortier-Davy M. Carhart-Harris R. Corlett P. R. Dupuis D. Halberstadt A. L. (2020). Hallucinations under psychedelics and in the schizophrenia spectrum: an interdisciplinary and multiscale comparison. *Schiz. Bull.* 46 1396–1408. 10.1093/schbul/sbaa117 32944778 PMC7707069

[B157] Letheby C. (2019). “The varieties of psychedelic epistemology,” in *Psychedelicacies: More Food for Thought From Breaking Convention*, eds Wyrd N. Luke D. Tollan A. Adams C. King D. (London: Strange Attractor Press).

[B158] Lifshitz M. Sheiner E. Kirmayer L. J. (2018). “Cultural neurophenomenology of psychedelic thought: guiding the “unconstrained” mind through ritual context,” in *The Oxford Handbook of Spontaneous Thought: Mind-Wandering, Creativity, and Dreaming*, eds Christoff L. Fox K. C. R. (Oxford: Oxford University Press), 573–594.

[B159] Lohmar D. (2010). “The function of weak phantasy in perception and thinking,” in *Handbook of Phenomenology and Cognitive Science*, eds Gallagher S. Schmicking D. (London: Springer), 159–177.

[B160] Lord L. D. Expert P. Atasoy S. Roseman L. Rapuano K. Lambiotte R. (2019). Dynamical exploration of the repertoire of brain networks at rest is modulated by psilocybin. *Neuroimage* 199 127–142. 10.1016/j.neuroimage.2019.05.060 31132450

[B161] Luke D. (2020). Anomalous psychedelic experiences: at the neurochemical juncture of the humanistic and parapsychological. *J. Hum. Psychol.* 10.1177/0022167820917767

[B162] Luoma J. B. Chwyl C. Bathje G. J. Davis A. K. Lancelotta R. (2020). A meta-analysis of placebo-controlled trials of psychedelic-assisted therapy. *J. Psychoactive Drugs* 52 289–299. 10.1080/02791072.2020.1769878 32529966 PMC7736164

[B163] Lutkajtis A. (2020). Entity encounters and the therapeutic effect of the psychedelic mystical experience. *J. Psychedelic Stud.* 4 171–178. 10.1556/2054.2020.00143

[B164] Ly C. Greb A. C. Cameron L. P. Wong J. M. Barragan E. V. Wilson P. C. (2018). Psychedelics promote structural and functional neural plasticity. *Cell Rep.* 23 3170–3182. 10.1016/j.celrep.2018.05.022 29898390 PMC6082376

[B165] MacDonald R. A. (2013). Music, health, and well-being: a review. *Int. J. Qual. Stud. Health Well Being* 8:20635. 10.3402/qhw.v8i0.20635 23930991 PMC3740599

[B166] MacLean K. A. Johnson M. W. Griffiths R. R. (2011). Mystical experiences occasioned by the hallucinogen psilocybin lead to increases in the personality domain of openness. *J. Psychopharmacol.* 25 1453–1461. 10.1177/0269881111420188 21956378 PMC3537171

[B167] Madsen M. K. Stenbæk D. S. Arvidsson A. Armand S. Marstrand-Joergensen M. R. Johansen S. S. (2021). Psilocybin-induced changes in brain network integrity and segregation correlate with plasma psilocin level and psychedelic experience. *Eur. Neuropsychopharmacol.* 50 121–132. 10.1016/j.euroneuro.2021.06.001 34246868

[B168] Mason N. L. Kuypers K. P. C. Müller F. Reckweg J. Tse D. H. Y. Toennes S. W. (2020). Me, myself, bye: regional alterations in glutamate and the experience of ego dissolution with psilocybin. *Neuropsychopharmacology* 45 2003–2011. 10.1038/s41386-020-0718-8 32446245 PMC7547711

[B169] Mason N. L. Kuypers K. P. C. Reckweg J. T. Müller F. Tse D. H. Y. Da Rios B. (2021). Spontaneous and deliberate creative cognition during and after psilocybin exposure. *Transl. Psychiatry* 11:209. 10.1038/s41398-021-01335-5 33833225 PMC8032715

[B170] Mason N. L. Mischler E. Uthaug M. V. Kuypers K. P. C. (2019). Sub-acute effects of psilocybin on empathy, creative thinking, and subjective well-being. *J. Psychoactive Drugs* 51 123–134. 10.1080/02791072.2019.1580804 30905276

[B171] McKay R. (2018). The role of experience in religion: accommodation vs. assimilation. *Relig. Brain Behav.* 8 428–431. 10.1080/2153599X.2017.1323782

[B172] McKenna T. (1992). *Food of the Gods. The Search for the Original Tree of Knowledge. A Radical History of Plants, Drugs, and Human Evolution.* New York, NY: Bantam Books.

[B173] McNamara P. (2009). *The Neuroscience of Religious Experience.* New York, NY: Cambridge University Press.

[B174] Mehr S. A. Singh M. Knox D. Ketter D. M. Pickens-Jones D. Atwood S. (2019). Universality and diversity in human song. *Science* 366 eaax0868. 10.1126/science.aax0868 31753969 PMC7001657

[B175] Miller M. J. Albarracin-Jordan J. Moore C. Capriles J. M. (2019). Chemical evidence for the use of multiple psychotropic plants in a 1,000-year-old ritual bundle from South America. *Proc. Natl. Acad. Sci. U.S.A.* 116 11207–11212. 10.1073/pnas.1902174116 31061128 PMC6561276

[B176] Milton K. (2003). The critical role played by animal source foods in human (*Homo*) evolution. *J. Nutr.* 133(11 Suppl 2) 3886S–3892S. 10.1093/jn/133.11.3886S 14672286

[B177] Miyazaki K. Miyazaki K. W. Doya K. (2012). The role of serotonin in the regulation of patience and impulsivity. *Mol. Neurobiol.* 45 213–224. 10.1007/s12035-012-8232-6 22262065 PMC3311865

[B178] Morgan T. J. H. (2016). Testing the cognitive and cultural niche theories of human evolution. *Curr. Anthropol.* 57 370–377. 10.1086/686531

[B179] Mueller F. Lenz C. Dolder P. Harder S. Schmid Y. Lang U. E. (2017). Acute effects of LSD on amygdala activity during processing of fearful stimuli in healthy subjects. *Transl. Psychiatry* 7 e1084. 10.1038/tp.2017.54 28375205 PMC5416695

[B180] Müller C. P. (2020). Drug instrumentalization. *Behav. Brain Res.* 15:112672. 10.1016/j.bbr.2020.112672 32442549

[B181] Müller C. P. Schumann G. (2011). Drugs as instruments: a new framework for non-addictive psychoactive drug use. *Behav. Brain Sci.* 34 293–347. 10.1017/S0140525X11000057 22074962

[B182] Munn H. (1973). “The mushrooms of language,” in *Hallucinogens and Shamanism*, ed. Harner M. J. (Oxford: Oxford University Press), 86–122.

[B183] Murnane K. S. (2018). The renaissance in psychedelic research: what do preclinical models have to offer. *Prog. Brain Res.* 242 25–67. 10.1016/bs.pbr.2018.08.003 30471682

[B184] Myerhoff B. G. (1974). *The Peyote Hunt: The Sacred Journey of the Huichol Indians.* New York, NY: Cornell University Press.

[B185] Nettl B. (1956). *Music in Primitive Culture.* Cambridge, MA: Harvard University Press. Available online at: 10.4159/harvard.9780674863408

[B186] Netzband N. Ruffell S. Linton S. Tsang W. Wolff T. (2020). Modulatory effects of ayahuasca on personality structure in a traditional framework. *Psychopharmacology* 237 1–11. 10.1007/s00213-020-05601-0 32700023 PMC7524857

[B187] Nichols D. E. (2004). Hallucinogens. *Pharmacol. Ther.* 101 131–181. 10.1016/j.pharmthera.2003.11.002 14761703

[B188] Nichols D. E. (2010). “Hallucinogens,” in *Encyclopedia of Psychopharmacology*, ed. Stolerman I. P. (Berlin: Springer), 10.1007/978-3-540-68706-1_44

[B189] Nichols D. E. (2016). Psychedelics. *Pharmacol. Rev.* 68 264–355. 10.1124/pr.115.011478 26841800 PMC4813425

[B190] Nielsen M. Langley M. C. Shiptonm C. Kapitány R. (2020). *Homo neanderthalensis* and the evolutionary origins of ritual in *Homo sapiens*. *Phil. Trans. R. Soc. B* 375:20190424. 10.1098/rstb.2019.0424 32594872 PMC7423259

[B191] Nilsson S. R. O. Phillips B. U. Sebastian F. A. Axelsson J. A. (2019). “Chapter eight - Serotonin and cognitive flexibility,” in *The Serotonin System*, eds Tricklebank M. D. Daly E. (Cambridge, MA: Academic Press), 133–154. 10.1016/B978-0-12-813323-1.00008-6

[B192] Noorani T. Alderson-Day B. (2020). Spotlight commentary: REBUS and the anarchic brain. *Neurosci. Consci.* 1:niaa007. 10.1093/nc/niaa007 32550008 PMC7290155

[B193] Norenzayan A. Shariff A. Gervais W. Willard A. McNamara R. Slingerland E. (2016). The cultural evolution of prosocial religions. *Behav. Brain Sci.* 39:E1. 10.1017/S0140525X14001356 26785995

[B194] Norenzyan A. (2010). “Why we believe: religion as a human universal,” in *Human Morality and Sociality: Evolutionary and Comparative Perspectives*, ed. Hogh-Oleson H. (New York, NY: Palgrave Macmillan), 58–71.

[B195] Nutt D. J. King L. A. Phillips L. D. (2010). Drug harms in the UK: a multicriteria decision analysis. *Lancet* 376 1558–1565. 10.1016/S0140-6736(10)61462-6 21036393

[B196] O’Brien M. Laland K. N. (2012). Genes, culture and agriculture: an example of human niche construction. *Curr. Anthropol.* 53 434–470. 10.1086/666585

[B197] Odling-Smee F. Laland K. N. Feldman M. W. (2003). *Niche Construction: The Neglected Process in Evolution.* New York, NY: Princeton University Press.

[B198] Oken B. S. (2008). Placebo effects: clinical aspects and neurobiology. *Brain* 131(Pt 11) 2812–2823. 10.1093/brain/awn116 18567924 PMC2725026

[B199] O’Regan H. J. Lamb A. L. Wilkinson D. M. (2016). The missing mushrooms: searching for fungi in ancient human dietary analysis. *J. Archaeol. Sci.* 75 139–143. 10.1016/j.jas.2016.09.009

[B200] Ott J. (1993). *Pharmacotheon: Entheogenic Drugs, Their Plant Sources and History.* Kennewick: Natural Products Co.

[B201] Passanisi A. Santo Di Nuovo L. Urgese C. P. (2015). The influence of musical expression on creativity and interpersonal relationships in children. *Proc. Soc. Behav. Sci.* 191 2476–2480. 10.1016/j.sbspro.2015.04.308

[B202] Pearce E. Launay J. Dunbar R. I. M. (2015). The ice-breaker effect: singing mediates fast social bonding. *R. Soc. Open Sci.* 2:150221. 10.1098/rsos.150221 26587241 PMC4632513

[B203] Pennacchio M. Jefferson L. V. Havens K. (2010). *Uses and Abuses of Plant-Derived Smoke: Its Ethnobotany as Hallucinogen, Perfume, Incense, and Medicine.* Oxford: Oxford University Press.

[B204] Peoples H. C. Duda P. Marlowe F. W. (2016). Hunter-gatherers and the origins of religion. *Hum. Nat.* 27 261–282. 10.1007/s12110-016-9260-0 27154194 PMC4958132

[B205] Petri G. Expert P. Turkheimer F. Carhart-Harris R. L. Nutt D. Hellyer P. J. (2014). Homological scaffolds of brain functional networks. *J. R. Soc. Interface* 11:20140873. 10.1098/rsif.2014.0873 25401177 PMC4223908

[B206] Pinker S. (2010). The cognitive niche: coevolution of intelligence, sociality, and language. *Proc. Natl. Acad. Sci. U.S.A.* 107 8993–8999. 10.1073/pnas.0914630107 20445094 PMC3024014

[B207] Pokorny T. Preller K. Kometer M. Dziobek I. Vollenweider F. X. (2017). Effect of psilocybin on empathy and moral decision-making. *Int. J. Neuropsychopharmacol.* 20 747–757. 10.1093/ijnp/pyx047 28637246 PMC5581487

[B208] Potts R. (2013). Hominin evolution in settings of strong environmental variability. *Q. Sci. Rev.* 73 1–13. 10.1016/j.quascirev.2013.04.003

[B209] Preller K. H. Vollenweider F. X. (2016). “Phenomenology, structure, and dynamic of psychedelic states,” in *Behavioral Neurobiology of Psychedelic Drugs. Current Topics in Behavioral Neurosciences 36*, eds Halberstadt A. L. Vollenweider F. X. Nichols D. E. (Berlin: Springer), 221–256. 10.1007/7854_2016_459 28025814

[B210] Preller K. H. Vollenweider F. X. (2019). Modulation of social cognition via hallucinogens and “entactogens”. *Front. Psychiatry* 10:881. 10.3389/fpsyt.2019.00881 31849730 PMC6902301

[B211] Preller K. H. Duerler P. Burt J. B. Ji J. L. Adkinson B. Stämpfli P. (2020). Psilocybin induces time-dependent changes in global functional connectivity. *Biol. Psychiatry* 88 197–207. 10.1016/j.biopsych.2019.12.027 32111343

[B212] Preller K. H. Razi A. Zeidman P. Stämpfli P. Friston K. J. Vollenweider F. X. (2019). Effective connectivity changes in LSD-induced altered states of consciousness in humans. *PNAS* 116 2743–2748. 10.1073/pnas.1815129116 30692255 PMC6377471

[B213] Puig M. V. Gulledge A. T. (2011). Serotonin and prefrontal cortex function: neurons, networks, and circuits. *Mol. Neurobiol.* 44 449–464. 10.1007/s12035-011-8214-0 22076606 PMC3282112

[B214] Quirce C. M. Badilla B. Badilla S. Martínez M. Rodríguez J. M. (2010). Los alucinógenos: su historia, antropología, química y farmacología [The hallucinogens: Their history, anthropology, chemistry, and pharmacology]. *Psicogente* 13 174–192.

[B215] Raghanti M. A. Edler M. K. Stephenson A. R. Munger E. L. Jacobs B. Hof P. R. (2018). A neurochemical hypothesis for the origin of hominids. *Proc. Natl. Acad. Sci. U.S.A.* 115 E1108–E1116. 10.1073/pnas.1719666115 29358369 PMC5819450

[B216] Rätsch C. (2005). *The Encyclopedia of Psychoactive Plants: Ethnopharmacology and its Applications.* Burlington, VT: Park Street Press.

[B217] Ray O. (2004). The revolutionary health science of psychoendoneuroimmunology: a new paradigm for understanding health and treating illness. *Ann. N. Y. Acad. Sci.* 1032 35–51. 10.1196/annals.1314.004 15677394

[B218] Reichel-Dolmatoff G. (1971). *Amazonian Cosmos: The Sexual & Religious Symbolism of the Tukano Indians.* Chicago, IL: University of Chicago Press.

[B219] Reiff C. M. Richman E. E. Nemeroff C. B. Carpenter L. L. Widge A. S. Rodriguez C. I. (2020). Psychedelics and psychedelic-assisted psychotherapy. *Am. J. Psychiatry* 177 391–410. 10.1176/appi.ajp.2019.19010035 32098487

[B220] Richerson P. J. Christiansen M. H. (Eds.) (2013). *Cultural Evolution: Society, Technology, Language, and Religion.* Cambridge, MA: The MIT Press.

[B221] Richerson P. J. Boyd R. Henrich J. (2010). Gene–culture coevolution in the age of genomics. *Proc. Natl. Acad. Sci. U.S.A.* 107 8985–8992. 10.1073/pnas.0914631107 20445092 PMC3024025

[B222] Rilling J. K. Sanfey A. G. (2011). The neuroscience of social decision-making. *Ann. Rev. Psychol.* 62 23–48. 10.1146/annurev.psych.121208.131647 20822437

[B223] Ripinsky-Naxon M. (1993). *The Nature of Shamanism: Substance and Function of a Religious Metaphor.* New York, NY: SUNY.

[B224] Rivier L. Lindgren J. E. (1972). “Ayahuasca,” the South American hallucinogenic drink: an ethnobotanical and chemical investigation. *Econ. Bot.* 26 101–129. 10.1007/BF02860772

[B225] Roberts P. Stewart B. A. (2018). Defining the ‘generalist specialist’ niche for Pleistocene *Homo sapiens*. *Nat. Hum. Behav.* 2 542–550. 10.1038/s41562-018-0394-4 31209320

[B226] Robinson D. W. Brown K. McMenemy M. Dennany L. Baker M. J. Allan P. (2020). Datura quids at Pinwheel Cave, California, provide unambiguous confirmation of the ingestion of hallucinogens at a rock art site. *Proc. Natl. Acad. Sci. U.S.A.* 117 31026–31037. 10.1073/pnas.2014529117 33229522 PMC7733795

[B227] Rocha J. Osório F. Crippa J. Bouso J. Rossi G. Hallak J. (2019). Serotonergic hallucinogens and recognition of facial emotion expressions: a systematic review of the literature. *Ther. Adv. Psychopharmacol.* 9:2045125319845774. 10.1177/2045125319845774 31065350 PMC6487767

[B228] Rodríguez E. Wrangham R. W. (1993). “Zoopharmacognosy. The use of medicinal plants by animals,” in *Phytochemical Potentials of Tropical Plants*, eds Downum K. R. Romeo J. T. Stafford H. A. (New York, NY: Plenum), 89–105.

[B229] Rodriguez E. Cavin J. C. West J. E. (1982). The possible role of Amazonian psychoactive plants in the chemotherapy of parasitic worms – a hypothesis. *J. Ethnopharmacol.* 6 303–309. 10.1016/0378-8741(82)90053-8 7154699

[B230] Rodríguez J. M. Quirce C. M. (2012). Las plantas y los hongos alucinógenos: reflexiones preliminares sobre su rol en la evolución humana [Hallucinogenic plants and mushrooms: Preliminary reflections on their role in human evolution]. *Rev. Reflexiones* 91 9–32.

[B231] Rossano M. (2007). Did meditating make us human? *Camb. Archaeol. J.* 17 47–58. 10.1017/S0959774307000054

[B232] Rossano M. (2009). Ritual behavior and the origins of modern cognition. *Camb. Archaeol. J.* 19 243–256. 10.1017/S0959774309000298

[B233] Rossano M. (2020). Ritual as resource management. *Phil. Trans. R. Soc. B* 375:20190429. 10.1098/rstb.2019.0429 32594870 PMC7423257

[B234] Sachs B. D. Ni J. R. Caron M. G. (2015). Brain 5-HT deficiency increases stress vulnerability and impairs antidepressant responses following psychosocial stress. *Proc. Natl. Acad. Sci. U.S.A.* 112 2557–2562. 10.1073/pnas.1416866112 25675490 PMC4345581

[B235] Samorini G. (2019). The oldest archaeological data evidencing the relationship of *Homo sapiens* with psychoactive plants: a worldwide overview. *J. Psychedelic Stud.* 3 63–80. 10.1556/2054.2019.008

[B236] Samorini G. (2020). “Mushroom effigies in archaeology: a methodological approach,” in *Fly Agaric. A Compendium of History, Pharmacology, Mythology, and Exploration*, ed. Feeney K. (Ellensburg, WA: Fly Agaric Press), 269–296.

[B237] Savage P. Loui P. Tarr B. Schachner A. Glowacki L. Mithen S. (2020). Music as a coevolved system for social bonding. *Behav. Brain Sci.* 1–36. 10.1017/S0140525X20000333 [Epub ahead of print]. 32814608

[B238] Sayers K. Lovejoy C. O. (2014). Blood, bulbs, and bunodonts: On evolutionary ecology and the diets of *Ardipithecus*, *Australopithecus*, and early *Homo*. *Q. Rev. Biol.* 89 319–357. 10.1086/678568 25510078 PMC4350785

[B239] Schmidt A. Kometer M. Bachmann R. Seifritz E. Vollenweider F. X. (2013). The NMDA antagonist ketamine and the 5-HT agonist psilocybin produce dissociable effects on structural encoding of emotional face expressions. *Psychopharmacology (Berl)* 225 227–239. 10.1007/s00213-012-2811-0 22836372

[B240] Schultes R. E. Hofmann A. Rätsch C. (2001). *Plants of the Gods: Their Sacred, Healing, and Hallucinogenic Powers.* Burlington, VT: Healing Arts Press.

[B241] Schultz J. C. (2002). Shared signals and the potential for phylogenetic espionage between plants and animals. *Integr. Comp. Biol.* 42 454–462. 10.1093/icb/42.3.454 21708739

[B242] Schwartz J. H. Tattersall I. (2015). Defining the genus *Homo*. *Science* 349 931–932. 10.1126/science.aac6182 26315422

[B243] Siegel R. K. (2005). *Intoxication: The Universal Drive for Mind-Altering Substances.* Rochester, NY: Park Street.

[B244] Sieroszewski W. L. (1900). *12 Lat w Kraju Jakutów [Twelve Years Among the Yakuts].* Warsaw: Nakł. F. Karpińskiego.

[B245] Sigafoos J. Green V. A. Edrisinha C. Lancioni G. E. (2007). Flashback to the 1960s: LSD in the treatment of autism. *Dev. Neurorehabilit.* 10 75–81. 10.1080/13638490601106277 17608329

[B246] Silk J. B. (2007). “Who lived in the environment of evolutionary adaptedness?,” in *The Evolution of Mind: Fundamental Questions and Controversies*, eds Gangestad S. W. Simpson J. A. (New York, NY: Guilford Press), 103–110.

[B247] Simonsson O. Sexton J. D. Hendricks P. S. (2021). Associations between lifetime classic psychedelic use and markers of physical health. *J. Psychopharmacol.* 35 447–452. 10.1177/0269881121996863 33719688 PMC8056715

[B248] Singh M. (2017). The cultural evolution of shamanism. *Behav. Brain Sci.* 41:e66. 10.1017/S0140525X17001893 28679454

[B249] Skoggard I. Ember C. R. Pitek E. Jackson J. C. Carolus C. (2020). Resource stress predicts changes in religious belief and increases in sharing behavior. *Hum. Nat.* 31 249–271. 10.1007/s12110-020-09371-8 32803730

[B250] Sobiecki J. F. (2002). A preliminary inventory of plants used for psychoactive purposes in southern African healing traditions. *Trans. R. Soc. South Afr.* 57 1–24. 10.1080/00359190209520523

[B251] Spitzer M. Thimm M. Hermle L. Holzmann P. Kovar K. A. Heimann H. (1996). Increased activation of indirect semantic associations under psilocybin. *Biol. Psychiatry* 39 1055–1057. 10.1016/0006-3223(95)00418-1 8780843

[B252] Spreng R. N. Andrews-Hanna J. R. (2015). “The default network and social cognition,” in *Brain Mapping*, ed. Toga A. W. (Cambridge, MA: Academic Press), 165–169. 10.1016/B978-0-12-397025-1.00173-1

[B253] St John G. (2006). Electronic dance music culture and religion: an overview. *Cult. Relig.* 7 1–25. 10.1080/01438300600625259

[B254] Stamets P. (1996). *Psilocybin Mushrooms of the World. An Identification Guide.* Berkeley, CA: Ten Speed Press.

[B255] Sterelny K. (2007). Social intelligence, human intelligence and niche construction. *Phil. Trans. R. Soc. Lond. B* 362 719–730. 10.1098/rstb.2006.2006 17255005 PMC2346527

[B256] Sterelny K. (2012). *The Evolved Apprentice: How Evolution Made Humans Unique.* Cambridge, MA: The MIT Press.

[B257] Sterelny K. (2014). “Constructing the cooperative niche,” in *Entangled Life. History, Philosophy and Theory of the Life Sciences*, Vol. 4 eds Barker G. Desjardins E. Pearce T. (Dordrecht: Springer), 10.1007/978-94-007-7067-6_13

[B258] Sterelny K. (2018). Religion re-explained. *Relig. Brain Behav.* 8 406–425. 10.1080/2153599X.2017.1323779

[B259] Stout D. Chaminade T. (2012). Stone tools, language and the brain in human evolution. *Philos. Trans. R. Soc. Lond. B Biol. Sci.* 367 75–87. 10.1098/rstb.2011.0099 22106428 PMC3223784

[B260] Strassman R. J. (1984). Adverse reactions to psychedelic drugs: a review of the literature. *J. Nervous Ment. Dis.* 172 577–595. 10.1097/00005053-198410000-00001 6384428

[B261] Strassman R. J. (2001). *DMT: The Spirit Molecule: A Doctor’s Revolutionary Research Into the Biology of Near-Death and Mystical Experiences.* Rochester, NY: Park Street Press.

[B262] Studerus E. Gamma A. Vollenweider F. X. (2010). Psychometric evaluation of the altered states of consciousness rating scale (OAV). *PLoS One* 5:e12412. 10.1371/journal.pone.0012412 20824211 PMC2930851

[B263] Studerus E. Kometer M. Hasler F. Vollenweider F. X. (2011). Acute, subacute and long-term subjective effects of psilocybin in healthy humans: a pooled analysis of experimental studies. *J. Psychopharmacol.* 25 1434–1452. 10.1177/0269881110382466 20855349

[B264] Sullivan R. J. Hagen E. H. (2002). Psychotropic substance-seeking: evolutionary pathology or adaptation? *Addiction* 97 389–400. 10.1046/j.1360-0443.2002.00024.x 11964056

[B265] Sullivan R. J. Hagen E. H. (2015). “Passive vulnerability or active agency? An evolutionarily ecological perspective of human drug use,” in *The Impact of Addictive Substances and Behaviours on Individual and Societal Well-Being*, eds Anderson P. Rehm J. Room R. (Oxford: Oxford University Press), 13–36.

[B266] Sullivan R. J. Hagen E. H. Hammerstein P. (2008). Revealing the paradox of drug reward in human evolution. *Proc. R. Soc. B* 275 1231–1241. 10.1098/rspb.2007.1673 18353749 PMC2367444

[B267] Sutton M. Q. Anderson E. N. (2010). *Introduction to Cultural Ecology*, 2nd Edn. Lanham: AltaMira Press.

[B268] Swanson L. R. (2018). Unifying theories of psychedelic drug effects. *Front. Pharmacol.* 9:1072. 10.3389/fphar.2018.00172 29568270 PMC5853825

[B269] Sweat N. W. Bates L. W. Hendricks P. S. (2016). The associations of naturalistic classic psychedelic use, mystical experience, and creative problem solving. *J. Psychoactive Drugs* 48 344–350. 10.1080/02791072.2016.1234090 27719438

[B270] Szabo A. (2019). “Effects of psychedelics on inflammation and immunity,” in *Advances in Psychedelic Medicine: State-of-the-Art Therapeutic Applications*, eds Winkelman M. J. Sessa B. (Santa Barbara, CA: ABC-CLIO), 193–213.

[B271] Tagliazucchi E. Roseman L. Kaelen M. Orban C. Muthukumaraswamy S. Murphy K. (2016). Increased global functional connectivity correlates with LSD-induced ego dissolution. *Curr. Biol.* 26 1043–1050. 10.1016/j.cub.2016.02.010 27085214

[B272] Tarr B. Launay J. Cohen E. Dunbar R. I. M. (2015). Synchrony and exertion during dance independently raise pain threshold and encourage social bonding. *Biol. Lett.* 11:20150767. 10.1098/rsbl.2015.0767 26510676 PMC4650190

[B273] Thompson C. Szabo A. (2020). Psychedelics as a novel approach to treating autoimmune conditions. *Immunol. Lett.* 228 45–54. 10.1016/j.imlet.2020.10.001 33035575

[B274] Timmermann C. Roseman L. Schartner M. Milliere R. Williams L. T. J. Erritzoe D. (2019). Neural correlates of the DMT experience assessed with multivariate EEG. *Sci. Rep.* 9:16324. 10.1038/s41598-019-51974-4 31745107 PMC6864083

[B275] Tishkoff S. A. Reed F. A. Ranciaro A. Voight B. F. Babbitt C. C. Silverman J. S. (2007). Convergent adaptation of human lactase persistence in Africa and Europe. *Nat. Genet.* 39 31–40. 10.1038/ng1946 17159977 PMC2672153

[B276] Tomasello M. (2014). *A Natural History of Human Thinking.* Cambridge, MA: Harvard University Press.

[B277] Tomasello M. Melis A. P. Tennie C. Wyman E. Herrmann E. (2012). Two key steps in the evolution of human cooperation: the interdependence hypothesis. *Curr. Anthropol.* 53 673–692. 10.1086/668207

[B278] Tooby J. Devore I. (1987). “The reconstruction of hominid behavioral evolution through strategic modeling,” in *The Evolution of Human Behavior: Primate Models*, ed. Kinzey W. G. (New York, NY: State University of New York Press), 183–237.

[B279] Tricklebank M. D. Daly E. (2019). *The Serotonin System: History, Neuropharmacology, and Pathology.* Amsterdam: Elsevier. 10.1016/C2016-0-04524-2

[B280] Turner V. W. (1964). “A ndembu doctor in practice,” in *Magic, Faith and Healing*, ed. Kiev A. (New York, NY: Free Press).

[B281] Turner V. W. (1969). *The Ritual Process: Structure and Anti-Structure.* Chicago, IL: Aldine Publishing Co.

[B282] Tylš F. Páleníček T. Horáček J. (2014). Psilocybin—Summary of knowledge and new perspectives. *Eur. Neuropsychopharmacol.* 24 342–356. 10.1016/j.euroneuro.2013.12.006 24444771

[B283] Tylš F. Palenicek T. Horacek J. (2016). “Neurobiology of the effects of psilocybin in relation to its potential therapeutic targets,” in *Neuropathology of Drug Addictions and Substance Misuse*, Vol. 2 ed. Preedy V. R. ( Cambridge, MA: Academic Press), 782–793. 10.1016/B978-0-12-800212-4.00073-X

[B284] Ungar P. S. Sponheimer M. (2011). The diets of early hominins. *Science* 334 190–193. 10.1126/science.1207701 21998380

[B285] Uthaug M. V. Mason N. L. Toennes S. W. Reckweg J. T. de Sousa Fernandes, Perna E. B. (2021). A placebo-controlled study of the effects of ayahuasca, set and setting on mental health of participants in ayahuasca group retreats. *Psychopharmacology* 238 1899–1910. 10.1007/s00213-021-05817-8 33694031 PMC8233273

[B286] van Amsterdam J. Opperhuizen A. van den Brink W. (2011). Harm potential of magic mushroom use: a review. *Regul. Toxicol. Pharmacol.* 59 423–429. 10.1016/j.yrtph.2011.01.006 21256914

[B287] van Ginneken V. van Meerveld A. Wijgerde T. Verheij E. de Vries E. van der Greef J. (2017). Hunter-prey correlation between migration routes of African buffaloes and early hominids: evidence for the “Out of Africa” hypothesis. *Integr. Mol. Med.* 4 1–5. 10.15761/imm.1000287

[B288] Varley T. F. Carhart-Harris R. Roseman L. Menon D. K. Stamatakis E. A. (2020). Serotonergic psychedelics LSD & psilocybin increase the fractal dimension of cortical brain activity in spatial and temporal domains. *Neuroimage* 220:117049. 10.1016/j.neuroimage.2020.117049 32619708

[B289] Veile A. (2018). Hunter-gatherer diets and human behavioral evolution. *Physiol. Behav.* 193 190–195. 10.1016/j.physbeh.2018.05.023 29800635

[B290] Villalba J. J. Provenza F. D. (2007). Self-medication and homeostatic behavior in herbivores: learning about the benefits of nature’s pharmacy. *Animal* 1 1360–1370. 10.1017/S1751731107000134 22444892

[B291] Vollenweider F. X. Preller K. H. (2020). Psychedelic drugs: neurobiology and potential for treatment of psychiatric disorders. *Nat. Rev. Neurosci.* 21 611–624. 10.1038/s41583-020-0367-2 32929261

[B292] Vollenweider F. X. Vontobel P. Hell D. Leenders K. L. (1999). 5-HT modulation of dopamine release in basal ganglia in psilocybin-induced psychosis in man: a PET study with [11C]raclopride. *Neuropsychopharmacology* 20 424–433. 10.1016/S0893-133X(98)00108-0 10192823

[B293] Walsh R. (1990). *The Spirit of Shamanism.* New York, NY: Tarcher/Putnam.

[B294] Warner R. (1980). Deception and self-deception in shamanism and psychiatry. *Int. J. Soc. Psychiatry* 26 41–52. 10.1177/002076408002600106 7399823

[B295] Watts R. Day C. Krzanowski J. Carhart-Harris R. (2017). Patients’ accounts of increased “connectedness” and “acceptance” after psilocybin for treatment resistant depression. *J. Hum. Psychol.* 57 520–564. 10.1177/0022167817709585

[B296] Weber B. H. Depew D. J. (Eds.) (2003). *Evolution and Learning: The Baldwin Effect Reconsidered.* Cambridge, MA: MIT Press.

[B297] Wei Y. de Lange S. C. Scholtens L. H. Watanabe K. Ardesch D. J. Jansen P. R. (2019). Genetic mapping and evolutionary analysis of human-expanded cognitive networks. *Nat. Commun.* 10:4839. 10.1038/s41467-019-12764-8 31649260 PMC6813316

[B298] White T. D. Asfaw B. Beyene Y. Haile-Selassie Y. Lovejoy C. O. Suwa G. (2009). Ardipithecus ramidus and the *Paleobiology of Early Hominid*s. *Science* 326 75–86. 10.1126/science.1175802 19810190

[B299] Whitehouse H. Lanman J. A. (2014). The ties that bind us: ritual, fusion, and identification. *Curr. Anthropol.* 55 674–695.

[B300] Whiten A. Erdal D. (2012). The human socio-cognitive niche and its evolutionary origins. *Philos. Trans. R. Soc. B* 367 2119–2129. 10.1098/rstb.2012.0114 22734055 PMC3385679

[B301] Wink M. van Wyk B. (2008). *Mind-Altering and Poisonous Plants of the World: A Scientifically Accurate Guide to 1200 Toxic and Intoxicating Plants.* Portland: Timber Press.

[B302] Winkelman M. J. (1992). *Shamans, Priests, and Witches. A Crosscultural Study of Magico-Religious Practitioners.* Tempe: Arizona State University.

[B303] Winkelman M. J. (2002). Shamanism and cognitive evolution. *Camb. Archaeol. J.* 12 71–101. 10.1017/S0959774302000045

[B304] Winkelman M. J. (2004). “Spirits as human nature and the fundamental structures of consciousness,” in *From Shaman to Scientist Essays on Humanity’s Search for Spirits*, ed. Houran J. (Lanham, MD: Scarecrow Press), 59–96.

[B305] Winkelman M. J. (2008). *Culture and Health: Applying Medical Anthropology.* San Francisco, CA: Jossey Bass/Wiley Publishers.

[B306] Winkelman M. J. (2009). Shamanism and the origins of spirituality and ritual healing. *J. Study Relig. Nat. Cult.* 34 458–489. 10.1558/JSRNC.V3I4.458

[B307] Winkelman M. J. (2010). *Shamanism: A Biopsychosocial Paradigm of Consciousness and Healing.* Santa Barbara, CA: ABC-CLIO.

[B308] Winkelman M. J. (2011a). “Shamanism and the alteration of consciousness,” in *Altering Consciousness Multidisciplinary Perspectives Volume 1: History, Culture and the Humanities*, eds Cardeña E. Winkelman M. J. (Santa Barbara, CA: Praeger ABC-CLIO), 159–180.

[B309] Winkelman M. J. (2011b). The shamanic paradigm: evidence from ethnology, neuropsychology and ethology. *Time Mind* 3 159–182. 10.2752/175169610X12632240392758

[B310] Winkelman M. J. (2013b). Shamanic cosmology as an evolutionary neurocognitive epistemology. *Int. J. Trans. Stud.* 32 79–99. 10.24972/IJTS.2013.32.1.79

[B311] Winkelman M. J. (2013a). Shamanism and psychedelics: a biogenetic structuralist paradigm of ecopsychology. *Eur. J. Ecopsychol.* 4 90–115.

[B312] Winkelman M. J. (2013c). “The integrative mode of consciousness: evolutionary origins of ecstasy,” in *Ekstasen: Kontexte – Formen – Wirkungen*, eds Passie T. Belschner W. Petrow E. (Würzburg: Ergon-Verlag), 67–83.

[B313] Winkelman M. J. (2015). Shamanism as a biogenetic structural paradigm for humans’ evolved social psychology. *Psychol. Relig. Spirituality* 7 267–277. 10.1037/rel0000034

[B314] Winkelman M. J. (2017). The mechanisms of psychedelic visionary experiences: hypotheses from evolutionary psychology. *Front. Neurosci.* 11:539. 10.3389/fnins.2017.00539 29033783 PMC5625021

[B315] Winkelman M. J. (2018). An ontology of psychedelic entity experiences in evolutionary psychology and neurophenomenology. *J. Psychedelic Stud.* 2 1–19. 10.1556/2054.2018.002

[B316] Winkelman M. J. (2019a). Introduction: evidence for entheogen use in pre-history and world religions. *J. Psychedelic Stud.* 3 43–62. 10.1556/2054.2019.024

[B317] Winkelman M. J. (2019b). “The evolutionary origins of the supernatural in ritual behaviors,” in *The Supernatural After the Neuro-Turn*, eds Craffert P. Baker J. Winkelman M. (London: Routledge), 48–68.

[B318] Winkelman M. J. (2019c). “The supernatural as innate cognitive operators,” in *The Supernatural After the Neuro- Turn*, eds Craffert P. Baker J. Winkelman M. (Londond: Routledge), 89–106.

[B319] Winkelman M. J. (2021c). The evolved psychology of psychedelic set and setting: inferences regarding the roles of shamanism and entheogenic ecopsychology. *Front. Pharmacol.* 12:619890. 10.3389/fphar.2021.619890 33732156 PMC7959790

[B320] Winkelman M. J. (2021a). A cross-cultural study of the elementary forms of religious life: shamanistic healers, priests, and witches. *Relig. Brain Behav.* 11 27–45. 10.1080/2153599X.2020.1770845

[B321] Winkelman M. J. (2021b). An ethnological analogy and biogenetic model for interpretation of religion and ritual in the past. *J. Archaeol. Method Theory* 10.1007/s10816-021-09523-9

[B322] Winkelman M. J. Hoffman M. (2015). “Hallucinogens and entheogens,” in *Vocubulary for the Study of Religion*, Vol. 2 eds Segal R. von Stuckrad K. (Leiden: Koninklijke Brill), 126–132.

[B323] Winkelman M. J. Sessa B. (Eds.) (2019). *Advances in psychedelic medicine.* Santa Barbara, CA: ABC-CLIO.

[B324] Wood R. M. Rilling J. K. Sanfey A. G. Bhagwagar Z. Rogers R. D. (2006). Effects of tryptophan depletion on the performance of an iterated prisoner’s dilemma game in healthy adults. *Neuropsychopharmacol* 31 1075–1084. 10.1038/sj.npp.1300932 16407905

[B325] Wrangham R. (2009). *Catching Fire: How Cooking Made us Human.* New York, NY: Basic Books.

[B326] Yaden D. Griffiths R. (2020). The subjective effects of psychedelics are necessary for their enduring therapeutic effects. *ACS Pharmacol. Transl. Sci.* 4 568–572. 10.1021/acsptsci.0c00194 33861219 PMC8033615

[B327] Young S. N. Leyton M. (2002). The role of serotonin in human mood and social interaction. Insight from altered tryptophan levels. *Pharmacol. Biochem. Behav.* 71 857–865. 10.1016/s0091-3057(01)00670-0 11888576

[B328] Zhang H. Greatrex R. (1987). *The Bowu zhi: An Annotated Translation.* Stockholm: Föreningen för Orientaliska Studier.

